# Predicting lack of clinical improvement following varicose vein ablation using machine learning

**DOI:** 10.1016/j.jvsv.2024.102162

**Published:** 2024-12-26

**Authors:** Ben Li, Naomi Eisenberg, Derek Beaton, Douglas S. Lee, Leen Al-Omran, Duminda N. Wijeysundera, Mohamad A. Hussain, Ori D. Rotstein, Charles de Mestral, Muhammad Mamdani, Graham Roche-Nagle, Mohammed Al-Omran

**Affiliations:** a Department of Surgery, University of Toronto, Toronto, ON, Canada; b Division of Vascular Surgery, St. Michael's Hospital, Unity Health Toronto, Toronto, ON, Canada; c Institute of Medical Science, University of Toronto, Toronto, ON, Canada; d Temerty Centre for Artificial Intelligence Research and Education in Medicine (T-CAIREM), University of Toronto, Toronto, ON, Canada; e Division of Vascular Surgery, Peter Munk Cardiac Centre, University Health Network, Toronto, ON, Canada; f Data Science & Advanced Analytics, Unity Health Toronto, University of Toronto, Toronto, ON, Canada; g Division of Cardiology, Peter Munk Cardiac Centre, University Health Network, Toronto, ON, Canada; h Institute of Health Policy, Management and Evaluation, University of Toronto, Toronto, ON, Canada; i ICES, University of Toronto, Toronto, ON, Canada; j School of Medicine, Alfaisal University, Riyadh, Saudi Arabia; k Department of Anesthesia, St. Michael's Hospital, Unity Health Toronto, Toronto, ON, Canada; l Li Ka Shing Knowledge Institute, St. Michael's Hospital, Unity Health Toronto, Toronto, ON, Canada; m Division of Vascular and Endovascular Surgery and the Center for Surgery and Public Health, Brigham and Women's Hospital, Harvard Medical School, Boston, MA; n Division of General Surgery, St. Michael's Hospital, Unity Health Toronto, Toronto, ON, Canada; o Leslie Dan Faculty of Pharmacy, University of Toronto, Toronto, ON, Canada; p Division of Vascular and Interventional Radiology, University Health Network, Toronto, ON, Canada; q Department of Surgery, King Faisal Specialist Hospital and Research Center, Riyadh, Saudi Arabia

**Keywords:** Machine learning, Prediction, Lack of clinical improvement, Varicose vein ablation

## Abstract

**Objective:**

Varicose vein ablation is generally indicated in patients with active/healed venous ulcers. However, patient selection for intervention in individuals without venous ulcers is less clear. Tools that predict lack of clinical improvement (LCI) after vein ablation may help guide clinical decision-making but remain limited. We developed machine learning (ML) algorithms that predict 1-year LCI after varicose vein ablation.

**Methods:**

The Vascular Quality Initiative database was used to identify patients who underwent endovenous or surgical varicose vein treatment for Clinical-Etiological-Anatomical-Pathophysiological (CEAP) C2 to C4 disease between 2014 and 2024. We identified 226 predictive features (111 preoperative [demographic/clinical], 100 intraoperative [procedural], and 15 postoperative [immediate postoperative course/complications]). The primary outcome was 1-year LCI, defined as a preoperative Venous Clinical Severity Score (VCSS) minus postoperative VCSS of ≤0, indicating no clinical improvement after vein ablation. The data were divided into training (70%) and test (30%) sets. Six ML models were trained using preoperative features with 10-fold cross-validation (Extreme Gradient Boosting [XGBoost], random forest, Naïve Bayes classifier, support vector machine, artificial neural network, and logistic regression). The primary model evaluation metric was area under the receiver operating characteristic curve (AUROC). The algorithm with the best performance was further trained using intraoperative and postoperative features. The focus was on preoperative features, whereas intraoperative and postoperative features were of secondary importance, because preoperative predictions offer the most potential to mitigate risk, such as deciding whether to proceed with intervention. Model calibration was assessed using calibration plots, and the accuracy of probabilistic predictions was evaluated with Brier scores. Performance was evaluated across subgroups based on age, sex, race, ethnicity, rurality, median Area Deprivation Index, prior ipsilateral varicose vein ablation, location of primary vein treated, and treatment type.

**Results:**

Overall, 33,924 patients underwent varicose vein treatment (30,602 endovenous [90.2%] and 3322 surgical [9.8%]) during the study period and 5619 (16.6%) experienced 1-year LCI. Patients who developed the primary outcome were older, more likely to be socioeconomically disadvantaged, and less likely to use compression therapy routinely. They also had less severe disease as characterized by lower preoperative VCSS, Varicose Vein Symptom Questionnaire scores, and CEAP classifications. The best preoperative prediction model was XGBoost, achieving an AUROC of 0.94 (95% confidence interval [CI], 0.93-0.95). In comparison, logistic regression had an AUROC of 0.71 (95% CI, 0.70-0.73). The XGBoost model had marginally improved performance at the intraoperative and postoperative stages, both achieving an AUROC of 0.97 (95% CI, 0.96-0.98). Calibration plots showed good agreement between predicted and observed event probabilities with Brier scores of 0.12 (preoperative), 0.11 (intraoperative), and 0.10 (postoperative). Of the top 10 predictors, 7 were preoperative features including VCSS, Varicose Vein Symptom Questionnaire score, CEAP classification, prior varicose vein ablation, thrombus in the greater saphenous vein, and reflux in the deep veins. Model performance remained robust across all subgroups.

**Conclusions:**

We developed ML models that can accurately predict outcomes after endovenous and surgical varicose vein treatment for CEAP C2 to C4 disease, performing better than logistic regression. These algorithms have potential for important utility in guiding patient counseling and perioperative risk mitigation strategies to prevent LCI after varicose vein ablation.

**Article Highlights**

• **Type of Research:** Machine learning (ML) based prognostic study using prospectively collected data from the Vascular Quality Initiative
• **Key Findings:** ML models were trained on 33,924 patients undergoing endovascular and surgical varicose vein treatment to predict 1-year lack of clinical improvement, achieving an area under the receiver operating characteristic curve of 0.94 (95% confidence interval, 0.93-0.95) with good calibration using preoperative data.
• **Take Home Message:** ML models can accurately predict lack of clinical improvement after varicose vein ablation and have potential to guide patient selection and periprocedural risk mitigation strategies.

Chronic venous disease leading to varicose veins affects >20% of the general population,^1^ with an elevated incidence in females.^2^ Varicose veins and their complications have an important impact on patients' quality of life^3^ and the associated economic burden of this disease exceeds $3 billion annually in the United States.^4^ For patients with Clinical-Etiological-Anatomical-Pathophysiological (CEAP) classification C5 (healed ulcer) and C6 (active ulcer) disease, vein ablation is accepted as the standard of care to improve ulcer healing time and decrease the risk of ulcer recurrence based on results from clinical trials and guidelines by the Society for Vascular Surgery (SVS), American Venous Forum, and American Vein and Lymphatic Society.^5^, ^6^, ^7^, ^8^ However, selection of patients with less severe disease (CEAP ≤C4) for vein ablation remains less clear given the higher potential for lack of clinical improvement (LCI).^9^ Rodríguez et al^9^ recently analyzed data from the Vascular Quality Initiative (VQI) varicose veins database and demonstrated that >15% of patients had LCI after vein ablation based on preoperative vs postoperative Venous Clinical Severity Scores (VCSS). Given the potential risks associated with vein ablation and the fact that patients often pay out of pocket for these procedures that may not be covered by insurance companies or universal health care systems, accurate prediction of LCI may allow for better patient selection and counselling to decrease the risk of unnecessary complications and financial burden on patients.^10^^,^^11^

Currently, there are no widely used tools to support prediction of LCI after varicose vein ablation. For example, the SVS VQI Cardiac Risk Index predicts outcomes after arterial, but not venous, interventions.^12^ Other tools such as the National Surgical Quality Improvement Program (NSQIP) online surgical risk calculator use modelling techniques that require manual input of clinical variables, which deters routine use in busy medical settings.^13^ With clinical judgment alone, the ability for clinicians to predict postprocedural outcomes is suboptimal, with previous studies demonstrating area under the receiver operating characteristic curve (AUROC) values ranging from 0.51 to 0.75.^14^ Therefore, there is an important need to develop better and more practical risk prediction tools for patients being considered for varicose vein ablation.

Machine learning (ML) is a rapidly advancing technology that allows computers to learn from data and make accurate predictions.^15^ Using advanced analytics, ML can model complex relationships between inputs (eg, patient characteristics) and outputs (eg, clinical outcomes).^15^ This field has been driven by the explosion of electronic information combined with increasing computational capabilities.^15^ The advantage of newer ML techniques over traditional statistical methods is that they can better model complex, multicollinear relationships between covariates and outcomes,^16^ which is common in health care data.^17^ Previously, ML was applied to the NSQIP database to develop an algorithm that predicts perioperative complications for >2900 distinct procedures.^18^ Given the heterogeneity of this cohort, better predictive performance may be achieved by building ML algorithms specific to patients undergoing varicose vein ablation using the VQI database, a dedicated vascular registry containing procedure-specific variables.^19^ We previously described ML algorithms trained on VQI data for predicting outcomes after aortic, carotid, and peripheral arterial interventions, which achieved superior performance compared with traditional statistical techniques such logistic regression and existing tools.^20^, ^21^, ^22^, ^23^, ^24^, ^25^ The development of a ML-based risk prediction algorithm for varicose vein ablation may complement these existing algorithms and expand clinical guidance for the management of patients with chronic venous disease. In this study, we used VQI data to develop ML algorithms that predict 1-year LCI after varicose vein ablation at the preoperative, intraoperative, and postoperative stages. The focus was on preoperative features; intraoperative and postoperative features were of secondary importance, because preoperative predictions offer the most potential to mitigate risk, such as deciding whether to proceed with intervention. We hypothesized that our ML models could achieve better predictive performance compared with logistic regression. Specifically, with a hypothesized AUROC of approximately 0.70 for logistic regression, an improvement in AUROC of >0.20 would be clinically significant based on existing literature.^9^^,^^26^

## Methods

### Study approval

The SVS Patient Safety Organization Research Advisory Council approved this project and provided the anonymized dataset. Patient consent was not required because the data originated from an anonymized registry.

### Design

This was a ML-based prognostic study with findings reported based on the Transparent Reporting of a Multivariable Prediction Model for Individual Prognosis or Diagnosis and Artificial Intelligence (TRIPOD+AI) statement.^27^

### Dataset

The VQI database is a clinical registry maintained by the SVS Patient Safety Organization with the goal of improving vascular care (www.vqi.org).^19^ Vascular surgeons, interventionalists, and other specialists across >1000 academic and community hospitals in the United States, Canada, and Singapore prospectively contribute demographic, clinical, and outcomes data on consecutive eligible vascular patients including information from their index procedure up to approximately 1 year of follow-up.^28^ Routine audits comparing submitted data to hospital claims are performed to ensure data accuracy.^29^

### Patient cohort

All patients who underwent endovenous or surgical varicose vein treatment from March 1, 2014, and January 2, 2024, in the VQI database were included. The VQI varicose vein registry captures varicose vein ablation procedures for CEAP ≥C2 venous disease performed in vein centers, office-based practices, ambulatory locations, and inpatient settings, including thermal radiofrequency ablation, thermal laser ablation, mechanochemical ablation, chemical ablation, embolic adhesive ablation, and open surgical treatment including high ligation, stripping, and phlebectomy.^30^ Patients with no reported preoperative or postoperative VCSS were excluded because the primary outcome of LCI requires both the preoperative and postoperative VCSS to be available. Patients with CEAP C5 or C6 disease were excluded because an active or healed ulcer is a clear indication for vein ablation to improve ulcer healing time and/or decrease the risk of ulcer recurrence.^5^, ^6^, ^7^, ^8^

### Features

Predictor variables (features) used in the ML models were divided into preoperative, intraoperative, and postoperative stages. Given the advantage of ML in handling many input features, all available VQI variables were used to maximize predictive performance. Preoperative features (n = 111) included demographics, medical history (including prior deep vein thrombosis, superficial phlebitis, varicose vein ablation, etc), clinical presentation (including CEAP classification, VCSS, Varicose Vein Symptom Questionnaire [VVSymQ] score, etc), and anatomical and physiological characteristics (including veins with reflux and/or thrombus on venous duplex ultrasound). Intraoperative features (n = 100) included type of anesthesia (none, sedation, local, tumescent, regional, general, etc), periprocedural anticoagulation, number of veins treated, location, maximum diameter, and length of the primary vein and other veins treated, treatment type, and specific technical aspects of endovenous and surgical treatments (including tip length for radiofrequency ablation, watts for laser ablation, chemical concentration and volume for foam sclerotherapy, volume for endovenous adhesive ablation, and number of phlebectomy incisions for surgical ablation, etc). Postoperative features (n = 15) included immediate postoperative complications before discharge (including allergic reaction, migraine, infection, pulmonary embolism, transient ischemic attack, and stroke), postoperative compression therapy, and plan for additional sclerotherapy. A complete list of features and their definitions can be found in Supplementary Tables I-III (online only).

## Outcome

The primary outcome was 1-year LCI, defined as a preoperative VCSS minus postoperative VCSS of ≤0. In other words, over a 1-year follow-up period, there was no decrease in VCSS after vein ablation, suggesting no evidence of clinical improvement. The VCSS is a validated physician-reported outcome tool used to measure the severity of venous disease.^31^ It involves a 30-point overall scoring system based on 10 components with a maximum score of 3 each, including pain, varicose veins, venous edema, skin pigmentation, inflammation, induration, active ulcer number, active ulcer duration, active ulcer size, and use of compression therapy.^31^ A higher score reflects greater disease severity.^31^ The VCSS is a dynamic and quantitative evaluation tool that is responsive to changes in venous disease severity and can assess treatment effects.^31^ This definition of LCI has been previously described by the Research Committee of the American Venous Forum and provides an objective, quantitative assessment of clinical improvement after vein ablation.^9^ This primary outcome was chosen because accurate prediction of LCI after vein ablation can provide clinicians with a better understanding of patients who may not benefit from vein ablation and, therefore, require appropriate counselling and/or consideration of other treatment approaches.

### Model development

We trained six different ML models to predict 1-year LCI after varicose vein ablation: Extreme Gradient Boosting (XGBoost), random forest, Naïve Bayes classifier, radial basis function support vector machine, multilayer perceptron artificial neural network with a single hidden layer, sigmoid activation function, and cross-entropy loss function, and logistic regression. These models were chosen based on their established efficacy in predicting postoperative outcomes using structured data.^32^, ^33^, ^34^ Logistic regression was selected as the baseline comparator to assess relative model performance because it is the most commonly applied statistical technique in traditional risk prediction tools.^35^

The data were divided randomly into training (70%) and testing (30%) sets. Testing data were reserved for model evaluation and not used for training to ensure fair model assessment. Random, rather than temporal, data splitting was performed owing to significant advances in endovascular vein ablation over the past few years.^36^^,^^37^ In this scenario, temporal splitting, whereby a model trained on older data is tested on newer data, would likely lead to an underestimation of predictive performance.^36^^,^^37^ To determine the optimal model hyperparameters, 10-fold cross-validation and grid search were applied to training data.^38^^,^^39^ Initial analysis demonstrated that the primary outcome occurred in 5619 of 33,924 patients (16.6%) in our cohort. To improve class balance, random oversample examples was applied to training data.^40^ Random oversample examples uses a smoothed bootstrapping approach to generate new samples from the feature space surrounding the minority class, a commonly used method to support predictive modelling of uncommon events.^40^ The models were then evaluated on test set data and ranked based on the primary discriminatory metric of AUROC. The models were compared at the preoperative stage because predictions at this timepoint offer the most potential to mitigate undesirable outcomes, such as deciding whether to proceed with intervention.^41^ Our best performing model was XGBoost, which had the following optimized hyperparameters: number of rounds = 250, maximum tree depth = 3, learning rate = 0.05, gamma = 0, column sample by tree = 0.9, minimum child weight = 1, and subsample = 0.9. A relatively low learning rate increases training time to improve model performance, while a relatively low number of training rounds and maximum tree depth reduce the risk of overfitting. Supplementary Table IV (online only) outlines the process for selecting these hyperparameters. Logistic regression with hyperparameter optimization using 10-fold cross-validation and grid search was used as the comparative model. All the predictor variables were added into the logistic regression model without transformations or feature selection with the goal of maximizing predictive performance of this reference model. In essence, this logistic regression model was optimized in the same fashion as the advanced ML algorithms.

After identifying the best performing ML model at the preoperative stage, we further trained the algorithm using intraoperative and postoperative data. This approach involved considering different features at each phase of the perioperative course. At the preoperative stage, only preoperative characteristics were considered. At the intraoperative stage, both preoperative and intraoperative features were used. At the postoperative stage, all preoperative, intraoperative, and postoperative features were inputted into the model. The approach allows clinicians to gain insights into a patient's risk at different stages of their perioperative course, thereby guiding decision-making before, during, and after an intervention. This model training method has been previously described, particularly for the development of prediction tools for long-term outcomes.^42^^,^^43^

### Statistical analysis

Preoperative, intraoperative, and postoperative features were summarized as means (standard deviation) or medians (interquartile range) for continuous variables and numbers (%) for categorical variables. Differences between patients with and without 1-year LCI were assessed using independent *t* tests (continuous variables) and χ^2^ tests (categorical variables). To account for multiple comparisons, Bonferroni correction was used to set statistical significance. *P* values were calculated to assess for statistical significance in baseline differences between groups and were not used to guide feature selection. Specifically, all available variables in the VQI database were used as input features for the ML models to maximize predictive performance. The primary model evaluation metric was AUROC (95% CI), a validated measurement of discriminatory ability that considers both sensitivity and specificity.^44^ ROC curves were presented in figure format to demonstrate model performance across a range of sensitivity and specificity thresholds. Secondary performance metrics were accuracy, sensitivity, specificity, positive predictive value (PPV), and negative predictive value (NPV). To assess model robustness, we plotted calibration curves and calculated Brier scores, a measurement of the agreement between predicted and observed event probabilities.^45^ In the final model, feature importance was determined by ranking the top 10 predictors based on variable importance scores (gain), a measurement of the relative importance of individual covariates in contributing to an overall prediction.^46^ Variable importance scores were calculated because they are directly relevant to XGBoost and included in the XGBoost R package.^47^ This built-in package allows for direct extraction of the relative importance of each input feature in generating an overall prediction, thereby facilitating model explainability.^47^ Variable importance scores for XGBoost are similar to Shapley value analysis for other ML models, both providing potential explanations for how individual features contribute to model predictions.^48^ Although Shapley value analysis has gained popularity in recent years,^48^ the built-in variable importance score analysis for XGBoost allows for a more efficient and direct analysis of feature importance for our best-performing model. Importantly, we did not perform feature selection based on feature importance owing to the unique advantage of advanced ML techniques in handling many input features, thereby maximizing potential predictive performance.^49^ There was no evidence of significant interactions between variables. Subgroup analysis was performed to assess feature importance in patients who underwent surgical treatment vs endovenous ablation. To assess model performance on various subpopulations, we evaluated predictive performance across demographic/clinical subgroups based on age, sex, race, ethnicity, rurality, median Area Deprivation Index (ADI) percentile, prior ipsilateral varicose vein ablation, location of primary vein treated, and treatment type. Model performance was also assessed on the last 2 years of data (2023 and 2024).

Based on a validated sample size calculator for clinical prediction models, to achieve a minimum AUROC of 0.7 with an outcome rate of approximately 16% and 111 preoperative features, a minimum sample size of 14,108 patients with 2258 events is required.^50^ Our cohort of 33,924 patients with 5619 primary events satisfied this sample size requirement. For variables of interest, missing data were <5%; hence, we adopted a complete-case analysis approach, considering only nonmissing covariates for each patient. This is a valid analytical method for datasets with minimal missing data (<5%) and reflects predictive modelling of real-world data, which inherently includes missing information.^51^^,^^52^ Patients lost to follow-up were censored. All analyses were conducted using R version 4.3.1.^53^

## Results

### Patients, events, and follow-up

From an initial cohort of 39,265 patients who underwent varicose vein ablation in the VQI database between March 1, 2014, and January 2, 2024, a total of 5341 patients were excluded for the following reasons: CEAP C6 (n = 1880), C5 (n = 1063), or C0 (n = 26) disease or no reported preoperative or postoperative VCSS (n = 2372). The final analysis included 33,924 patients who underwent varicose vein treatment, including 30,602 endovenous (90.2%) and 3322 open (9.8%) surgical procedures. Overall, 5619 patients (16.6%) experienced 1-year LCI. Mean follow-up was 14.1 ± 1.1 months, and 31,040 patients (91.5%) had a follow-up time of ≥1 year.

### Preoperative characteristics

Compared with patients without a primary outcome, those who developed 1-year LCI were older, had a higher body mass index, and were more likely to be Black, receive Medicare or Medicaid, and have a higher median ADI percentile indicating greater socioeconomic disadvantage. There was no statistically significant difference in self-pay insurance status between patients with vs without 1-year LCI; however, rates of self-pay were relatively low in the cohort (<1%). These patients were also more likely to have a history of bilateral deep vein thrombosis, superficial phlebitis, and varicose vein ablation. Preoperative anticoagulation was more common in patients with 1-year LCI as prophylaxis or treatment for venous thromboembolism, arrhythmia management, or other clinical reason. Patients with 1-year LCI were more likely to have a CEAP classification of C2 as opposed to C3 or C4, indicating less severe venous disease. They also had a lower mean preoperative VCSS (5.79 ± 2.35 vs 7.29 ± 2.56; *P* < .001), driven by lower scores in the pain, varicose veins, venous edema, skin pigmentation, inflammation, induration, and use of compression therapy categories. Similarly, mean preoperative VVSymQ scores were lower in the 1-year LCI group (8.10 ± 5.59 vs 8.79 ± 5.53; *P* < .001), driven by lower scores in the heaviness, achiness, and throbbing categories. Patients with 1-year LCI were more likely to have reflux in the deep veins and thrombus in the greater saphenous vein (GSV) of the limb treated (Table I).

**Table I:** Preoperative demographic and clinical characteristics of patients undergoing varicose vein ablation with and without 1-year clinical improvement

	1-Year clinical improvement (n = 28,305)	1-Year LCI (n = 5619)	*P* value
Demographics			
Age, years, mean ± SD	55.0 ± 13.8	55.6 ± 14.1	.004
Female sex	20,232 (71.5)	4000 (71.2)	.90
BMI, kg/m^2^, mean ± SD	29.4 ± 6.8	29.8 ± 7.2	<.001
Race			
American Indian or Alaskan Native	48 (0.2)	10 (0.2)	.004
Asian	391 (1.4)	77 (1.4)	
Black	918 (3.2)	247 (4.4)	
Native Hawaiian or other Pacific Islander	28 (0.1)	5 (0.1)	
White	23,655 (83.6)	4646 (82.7)	
More than one race	73 (0.3)	11 (0.2)	
Unknown/other	3192 (11.3)	623 (11.1)	
Hispanic ethnicity	2006 (7.1)	328 (5.8)	<.001
Insurance status			
Medicare	6051 (21.4)	1298 (23.1)	<.001
Medicaid	1542 (5.5)	369 (6.6)	
Commercial	20,002 (70.7)	3852 (68.6)	
Military/Veterans Affairs	411 (1.5)	54 (1.0)	
Non-US insurance	8 (0.03)	0	
Self-pay (uninsured)	290 (1.0)	46 (0.8)	
Unknown/other	1 (0.004)	0	
Rural residence	343 (1.2)	54 (1.0)	.13
ADI percentile, median (IQR)	48 (47-49)	50 (49-51)	<.001
Medical history			
Prior deep vein thrombosis			
Ipsilateral	840 (3.0)	164 (2.9)	.004
Contralateral	437 (1.5)	81 (1.4)	
Bilateral	208 (0.7)	68 (1.2)	
Side not reported	106 (0.4)	14 (0.2)	
Prior superficial phlebitis			
Ipsilateral	1826 (6.5)	346 (6.2)	<.001
Contralateral	643 (2.3)	153 (2.7)	
Bilateral	417 (1.5)	124 (2.2)	
Side not reported	64 (0.2)	8 (0.1)	
Prior pulmonary embolism	177 (0.6)	20 (0.4)	.02
Prior varicose vein ablation			
Ipsilateral	3751 (13.3)	677 (12.0)	<.001
Contralateral	4605 (16.3)	878 (15.6)	
Bilateral	5365 (19.0)	1263 (22.5)	
No. of pregnancies, median (IQR)	3 (0-7)	2 (0-6)	<.001
Preoperative anticoagulation	1628 (5.8)	457 (8.1)	<.001
Prophylactic for venous thromboembolism	174 (0.6)	43 (0.8)	<.001
Therapeutic for venous thromboembolism	107 (0.4)	40 (0.7)	
Arrhythmia	194 (0.7)	72 (1.3)	
Other reason	1153 (4.1)	302 (5.4)	
Clinical presentation			
CEAP classification			
C2 (varicose veins)	10,176 (36.0)	2284 (40.6)	<.001
C2r (recurrent varicose veins)	172 (0.6)	35 (0.6)	
C3 (edema)	11,539 (40.8)	2284 (40.6)	
C4a (pigmentation or eczema)	5440 (19.2)	899 (16.0)	
C4b (lipodermatosclerosis or atrophie blanche)	943 (3.3)	116 (2.1)	
C4c (corona phlebectatica)	35 (0.1)	1 (0.02)	
VCSS, mean ± SD, maximum score = 30 (10 components with maximum score of 3 each), higher score means greater severity	7.29 ± 2.56	5.79 ± 2.35	<.001
Pain, mean ± SD	1.79 ± 0.71	1.38 ± 0.70	<.001
Varicose veins, mean ± SD	2.05 ± 0.74	1.69 ± 0.83	<.001
Venous edema, mean ± SD	0.88 ± 0.48	0.72 ± 0.47	<.001
Skin pigmentation, mean ± SD	0.30 ± 0.14	0.24 ± 0.19	<.001
Inflammation, mean ± SD	0.20 ± 0.12	0.12 ± 0.09	<.001
Induration, mean ± SD	0.13 ± 0.09	0.07 ± 0.03	<.001
Active ulcer number, mean ± SD	0 ± 0	0 ± 0	.99
Active ulcer duration, mean ± SD	0 ± 0	0 ± 0	.99
Active ulcer size, mean ± SD	0 ± 0	0 ± 0	.99
Use of compression therapy, mean ± SD	1.97 ± 1.04	1.59 ± 1.11	<.001
VVSymQ score, mean ± SD, maximum score = 25 (5 components with maximum score of 5 each), higher score means more symptomatic and/or greater impact on patients	8.79 ± 5.53	8.10 ± 5.59	<.001
Heaviness, mean ± SD	1.94 ± 1.53	1.69 ± 1.52	<.001
Achiness, mean ± SD	2.32 ± 1.42	2.05 ± 1.46	<.001
Swelling, mean ± SD	1.92 ± 1.17	1.89 ± 1.11	.15
Throbbing, mean ± SD	1.52 ± 1.00	1.40 ± 1.02	<.001
Itching, mean ± SD	1.09 ± 0.39	1.07 ± 0.34	.34
Appearance, median ± SD, maximum score = 4, higher score means more noticeable to patients	2.28 ± 1.27	2.25 ± 1.34	.13
Work impact, median ± SD, maximum score = 5, higher score means greater reduction in work activity	1.71 ± 1.26	1.45 ± 1.27	<.001
Anatomical and physiological characteristics			
Imaging			
Duplex ultrasound	28,005 (98.9)	5523 (98.3)	<.001
Other	19 (0.07)	7 (0.1)	
Reflux on duplex ultrasound >0.5 seconds			
GSV thigh	18,126 (64.0)	3734 (66.5)	<.001
GSV calf	14,224 (50.3)	2511 (44.7)	<.001
Anterior accessory GSV thigh	5290 (18.7)	824 (14.7)	<.001
Anterior accessory GSV calf	2162 (7.6)	76 (1.4)	<.001
Superficial accessory GSV	753 (2.7)	18 (0.3)	<.001
GSV remnant	971 (3.4)	231 (4.1)	.01
Small saphenous vein	7854 (27.7)	1396 (24.8)	<.001
Deep veins	8289 (29.3)	2518 (44.8)	<.001
Other veins^a^	8261 (29.2)	702 (12.5)	<.001
Thrombus seen on duplex ultrasound			
GSV thigh	391 (1.4)	134 (2.4)	<.001
GSV calf	295 (1.0)	87 (1.6)	.001
Anterior accessory GSV thigh	64 (0.2)	21 (0.4)	.06
Anterior accessory GSV calf	21 (0.07)	5 (0.09)	.92
Superficial accessory GSV	26 (0.09)	0	.04
GSV remnant	16 (0.06)	8 (0.1)	.053
Small saphenous vein	315 (1.1)	72 (1.3)	.31
Deep veins	202 (0.7)	48 (0.9)	.30
Other veins^a^	107 (0.4)	10 (0.2)	.03

*ADI,* Area Deprivation Index; *BMI,* Body mass index; *CEAP,* Clinical-Etiological-Anatomical-Pathophysiological; *GSV,* great saphenous vein; *IQR,* interquartile range; *LCI,* lack of clinical improvement; *SD,* standard deviation; *VCSS,* Venous Clinical Severity Score; *VVSymQ,* Varicose Vein Symptom Questionnaire.

Values are reported as number (%) unless otherwise indicated.

a Other veins include posterior accessory great saphenous vein, anterior thigh perforating vein, posterior thigh circumflex vein, intersaphenous veins including the Giacomini vein, lateral thigh veins, perforating veins in the thigh, perforating veins at the knee, perforating veins in the calf, and perforating veins at the ankle.

### Intraoperative characteristics

Patients with 1-year LCI were more likely to receive local anesthesia and less likely to receive sedation or general anesthesia. The differences in the type of anesthesia between groups likely reflect the type of procedures they underwent (eg, surgical vein stripping vs endovenous ablation). Periprocedural prophylactic anticoagulation with low-molecular-weight heparin was more common in the 1-year LCI group. Patients with a primary outcome had a lower median number of veins treated and were more likely to have a truncal vein treated, including the GSV and small saphenous vein. Their primary vein treated had a longer median length with no difference in the median diameter. Patients with 1-year LCI were more likely to undergo open surgical, rather than endovenous, treatment. If they underwent foam sclerotherapy, they were more likely to receive carbon dioxide gas and a higher concentration and greater volume of foam. If they underwent endovenous adhesive ablation, they received a lower mean volume of embolic adhesive. The number of phlebectomy incisions was lower in patients with 1-year LCI, which may reflect the fact that patients with lower varicose vein burden preoperatively were more likely to have 1-year LCI (Table II).

**Table II:** Intraoperative characteristics of patients undergoing varicose vein ablation with and without 1-year clinical improvement

	1-Year clinical improvement (n = 28,305)	1-Year LCI (n = 5619)	*P* value
Anesthesia			
None	3746 (13.2)	645 (11.5)	<.001
Minimal sedation	6084 (21.5)	845 (15.0)	<.001
Moderate sedation	1628 (5.8)	120 (2.1)	<.001
Deep sedation	861 (3.0)	124 (2.2)	<.001
Local	14,567 (51.5)	3450 (61.4)	<.001
Tumescent	20,074 (70.9)	3724 (66.3)	<.001
Regional	15 (0.05)	2 (0.04)	.84
General	3542 (12.5)	338 (6.0)	<.001
Periprocedural anticoagulation			
Low-molecular-weight heparin	973 (3.4)	630 (11.2)	<.001
Unfractionated heparin	1188 (4.2)	198 (3.5)	
Other	373 (1.3)	89 (1.6)	
No. of veins treated, median (IQR)	2 (1-2)	1 (1-2)	<.001
Location of primary vein treated			
Truncal	21,207 (74.9)	4454 (79.3)	<.001
Truncal, recanalized/remnant	754 (2.7)	308 (5.5)	
Cluster (superficial varicosities)	5975 (21.1)	791 (14.1)	
Perforator	344 (1.2)	63 (1.1)	
Perforator, recanalized	5 (0.02)	3 (0.05)	
Other	20 (0.1)	0	
Truncal vein treated			
GSV thigh and calf	6948 (24.5)	1093 (19.5)	<.001
GSV thigh	7724 (27.3)	2167 (38.6)	
GSV calf	2151 (7.6)	577 (10.3)	
Superficial accessory GSV thigh	110 (0.4)	9 (0.2)	
Anterior accessory GSV thigh	1755 (6.2)	325 (5.8)	
Anterior accessory GSV calf	247 (0.9)	14 (0.2)	
Small saphenous vein thigh extension	102 (0.4)	16 (0.3)	
Small saphenous vein calf	1901 (6.7)	428 (7.6)	
Other	1012 (3.6)	126 (2.2)	
Perforator veins treated			
Thigh	107 (0.4)	25 (0.4)	.44
Calf	224 (0.8)	40 (0.7)	
Ankle	18 (0.06)	1 (0.02)	
Maximum diameter of primary vein treated, mm, median (IQR)	6.5 (5.0-8.3)	6.4 (5.0-8.0)	.10
Length of primary vein treated, cm, median (IQR)	34.0 (26.5-41.0)	36.0 (28.0-45.0)	<.001
Treatment type			
Thermal, radiofrequency ablation	12,056 (42.6)	3084 (54.9)	<.001
Thermal, laser	7917 (28.0)	945 (16.8)	
Surgery	2736 (9.7)	586 (10.4)	
Other endovenous	5596 (19.8)	1004 (17.9)	
Tip length for radiofrequency ablation			
2.5 cm	7 (0.02)	0	<.001
3.0 cm	584 (2.1)	78 (1.4)	
7.0 cm	11,098 (39.2)	2983 (53.1)	
10.0 cm	84 (0.3)	3 (0.05)	
Radiofrequency stylet for perforator veins	19 (0.07)	9 (0.2)	
Other	15 (0.05)	0	
Watts for laser ablation, mean ± SD	7.25 ± 1.24	7.41 ± 1.10	<.001
Foam sclerotherapy gas			
Air	3107 (11.0)	226 (4.0)	<.001
Carbon dioxide	369 (1.3)	331 (5.9)	
Other	2 (0.007)	1 (0.02)	
Chemical concentration for foam sclerotherapy, %, mean ± SD	4.76 ± 1.97	5.12 ± 1.44	<.001
Volume of foam injected, mL, mean ± SD	4.77 ± 1.98	5.13 ± 145	<.001
Volume of embolic adhesive injected, mL, mean ± SD	1.60 ± 0.17	1.50 ± 0.11	<.001
No. of phlebectomy incisions			
<10	885 (3.1)	193 (3.4)	.002
10-20	1401 (5.0)	324 (5.8)	
>20	958 (3.4)	149 (2.7)	

*GSV,* Great saphenous vein; *IQR,* interquartile range; *LCI,* lack of clinical improvement; *SD,* standard deviation.

Values are reported as number (%) unless otherwise indicated.

### Postoperative characteristics

Immediate postoperative complications, including allergic reaction, migraine, infection, pulmonary embolism, transient ischemic attack, stroke, and death, were rare (<0.3%) in the cohort, with no differences between patients with and without 1-year LCI. Patients with 1-year LCI were more likely to be prescribed a postoperative compression stocking and less likely to be prescribed a compression bandage. They also had a lower mean number of days of compression therapy prescribed (8.69 ± 2.29 days vs 9.16 ± 3.65 days; *P* < .001) and were more likely to have additional sclerotherapy planned for the ipsilateral leg (4.2% vs 2.9%; *P* < .001) (Table III).

**Table III:** Immediate postoperative characteristics and complications of patients undergoing varicose vein ablation with and without 1-year clinical improvement

	1-Year clinical improvement (n = 28,305)	1-Year LCI (n = 5619)	*P* value
Complications			
Mild allergic reaction requiring local treatment	12 (0.04)	3 (0.05)	.99
Severe allergic reaction requiring systemic treatment	1 (0.004)	0	.99
Migraine	7 (0.02)	0	.50
Visual disturbance	5 (0.02)	0	.69
Cough or chest tightness	2 (0.007)	0	.99
Systemic infection	1 (0.004)	1 (0.02)	.75
Pulmonary embolism	3 (0.01)	1 (0.02)	.99
Transient ischemic attack	0	0	.99
Stroke	1 (0.004)	0	.99
Other complication	32 (0.1)	4 (0.07)	.51
Hospital admission required			
Unplanned	33 (0.1)	7 (0.1)	.50
Planned	5 (0.02)	1 (0.02)	
Postoperative compression therapy			
Stocking	10,961 (38.7)	2314 (41.2)	<.001
Bandage	6229 (22.0)	1096 (19.5)	
Stocking and/or bandage	10,380 (36.7)	2033 (36.2)	
None or not reported	735 (2.6)	176 (3.1)	
No. of days of compression therapy prescribed, mean ± SD	9.16 ± 3.65	8.69 ± 2.29	<.001
Additional sclerotherapy planned for ipsilateral leg	828 (2.9)	237 (4.2)	<.001

*LCI,* Lack of clinical improvement; *SD,* standard deviation.

Values are reported as number (%) unless otherwise indicated.

### Model performance

Of the six ML models evaluated at the preoperative stage using test set data, XGBoost had the best performance in predicting 1-year LCI (AUROC, 0.94; 95% CI, 0.93-0.95). In comparison, the other models had the following AUROCs: random forest (0.86; 95% CI, 0.85–0.87), radial basis function support vector machine (0.83; 95% CI, 0.81-0.84), Naïve Bayes (0.77; 95% CI, 0.76-0.79), multilayer perceptron artificial neural network (0.74; 95% CI, 0.73-0.75), and logistic regression (0.71; 95% CI, 0.70-0.73). The secondary performance metrics of XGBoost were the following: accuracy 0.85 (95% CI, 0.84-0.86), sensitivity 0.87, specificity 0.84, PPV 0.83, and NPV 0.87. Model performance results are summarized in Table IV.

**Table IV:** Model performance on test set data for predicting 1-year lack of clinical improvement (LCI) after varicose vein ablation using preoperative features

	AUROC (95% CI)	Accuracy (95% CI)	Sensitivity (95% CI)	Specificity (95% CI)	PPV (95% CI)	NPV (95% CI)
XGBoost	0.94 (0.93-0.95)	0.85 (0.84-0.86)	0.87 (0.86-0.88)	0.84 (0.83-0.85)	0.83 (0.82-0.84)	0.87 (0.86-0.88)
Random forest	0.86 (0.85-0.87)	0.78 (0.77-0.80)	0.78 (0.77-0.80)	0.79 (0.78-0.80)	0.80 (0.79-0.81)	0.77 (0.75-0.78)
RBF SVM	0.83 (0.81-0.84)	0.75 (0.74-0.77)	0.77 (0.76-0.78)	0.74 (0.73-0.75)	0.74 (0.72-0.75)	0.77 (0.76-0.79)
Naïve Bayes	0.77 (0.76-0.79)	0.72 (0.71-0.73)	0.73 (0.72-0.74)	0.71 (0.70-0.72)	0.71 (0.69-0.72)	0.73 (0.72-0.74)
MLP ANN	0.74 (0.73-0.75)	0.69 (0.68-0.71)	0.71 (0.69-0.73)	0.68 (0.67-0.70)	0.67 (0.66-0.68)	0.72 (0.71-0.73)
Logistic regression	0.71 (0.70-0.73)	0.64 (0.62-0.65)	0.60 (0.59-0.62)	0.73 (0.71-0.72)	0.64 (0.63-0.65)	0.62 (0.61-0.63)

*AUROC,* Area under the receiver operating characteristic curve; *CI,* confidence interval; *MLP ANN,* multilayer perceptron artificial neural network; *NPV,* negative predictive value; *PPV,* positive predictive value; *RBF SVM,* radial basis function support vector machine; *XGBoost,* Extreme Gradient Boosting.

The sample size denominators are the following: accuracy (n = 33,924), sensitivity (n = 5619), and specificity (n = 28,305).

**Fig 1:**
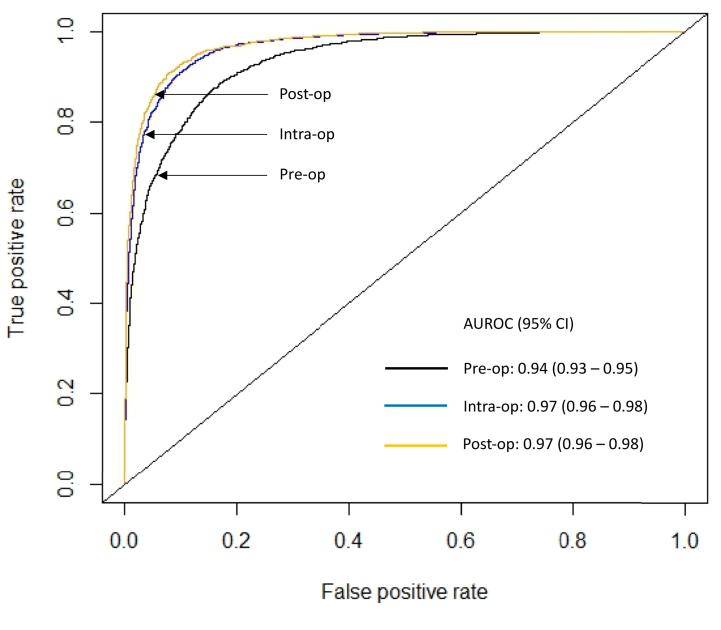
Receiver operating characteristic curve for predicting 1-year lack of clinical improvement after varicose vein ablation using Extreme Gradient Boosting (XGBoost) models at the preoperative, intraoperative, and postoperative stages. Secondary performance metrics: Preoperative: AUROC 0.94 (0.93-0.95), accuracy 0.85 (95% CI, 0.84-0.86), sensitivity 0.87, specificity 0.84, positive predictive value (PPV) 0.83, negative predictive value (NPV) 0.87. Intraoperative: AUROC 0.97 (0.96-0.98), accuracy 0.91 (95% CI, 0.90-0.92), sensitivity 0.92, specificity 0.89, PPV 0.89, NPV 0.92. Postoperative: AUROC 0.97 (0.96-0.98), accuracy 0.91 (95% CI, 0.90-0.92), sensitivity 0.93, specificity 0.90, PPV 0.90, NPV 0.93. *AUROC*, area under the receiver operating characteristic curve; *CI*, confidence interval.

**Fig 2:**
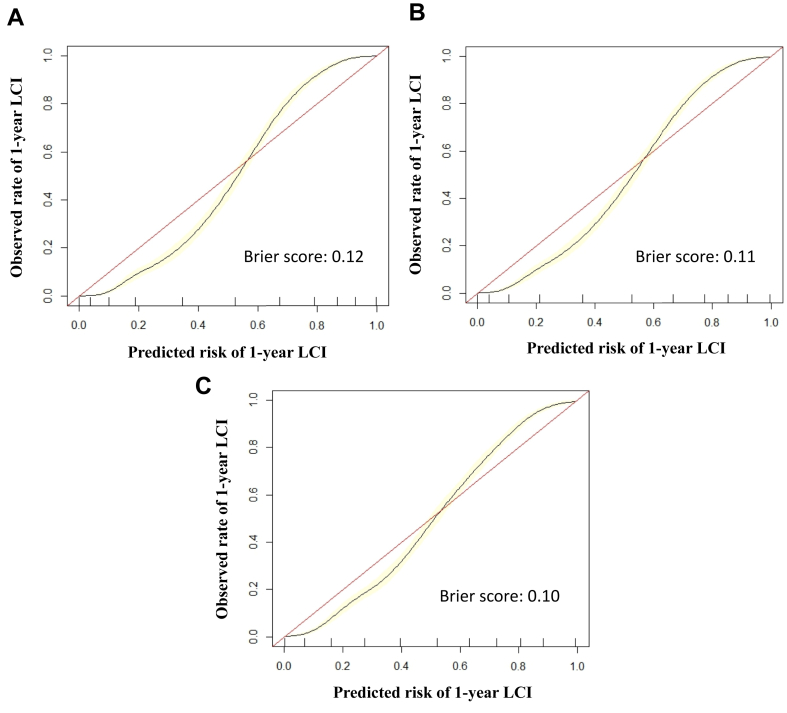
Calibration plots with Brier scores for predicting 1-year lack of clinical improvement (*LCI*) after varicose vein ablation using Extreme Gradient Boosting (XGBoost) models at the **(A)** preoperative, **(B)** intraoperative, and **(C)** postoperative stages.

We further trained the XGBoost model using intraoperative and postoperative data. The addition of intraoperative features improved performance to an AUROC of 0.97 (95% CI, 0.96-0.98). Adding postoperative features did not further change model performance, with the AUROC remaining at 0.97 (95% CI, 0.96-0.98). The ROC curves are presented in Fig 1. There was good agreement between predicted and observed event probabilities as demonstrated by the calibration plots in Fig 2, *A*-*C*, with Brier scores of 0.12 (preoperative), 0.11 (intraoperative), and 0.10 (postoperative).

**Fig 3:**
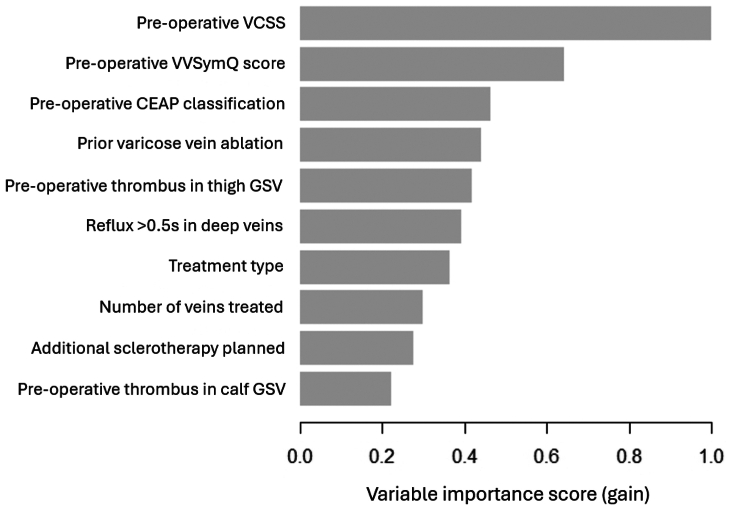
Variable importance scores (gain) for the top 10 predictors of 1-year lack of clinical improvement (LCI) after varicose vein ablation in the Extreme Gradient Boosting (XGBoost) model at the postoperative stage. *CEAP*, Clinical-Etiological-Anatomical-Pathophysiological; *GSV*, great saphenous vein; *VCSS*, Venous Clinical Severity Score; *VVSymQ*, Varicose Vein Symptom Questionnaire.

The top 10 predictors of 1-year LCI in the final XGBoost model included 7 preoperative features (VCSS, VVSymQ score, CEAP classification, prior varicose vein ablation, thrombus in the thigh GSV, reflux >0.5 second in deep veins, and thrombus in the calf GSV), 2 intraoperative features (treatment type and number of veins treated), and 1 postoperative feature (additional sclerotherapy planned) (Fig 3). To account for the long period of recruitment, we included procedure year as an input feature. We did not find procedure year to be a significant predictor of 1-year LCI. On subgroup analysis based on treatment type, 8 of the top 10 predictors were the same for patients who underwent surgical vs endovascular treatment, with the top 5 features being preoperative VCSS, VVSymQ score, CEAP classification, prior varicose vein ablation, and thrombus in the thigh GSV for both groups (Supplementary Fig 1, online only).

### Subgroup analysis

Model performance remained robust on subgroup analyses across demographic/clinical populations based on age, sex, race, ethnicity, rurality, median ADI percentile, prior ipsilateral varicose vein ablation, location of primary vein treated, and treatment type, with AUROC's ranging from 0.93-0.95 and no significant differences between majority and minority groups (Supplementary Figs 2-10, online only). Additionally, model performance remained excellent on the last 2 years of data (2023 and 2024), with an AUROC of 0.94 (95% CI, 0.93-0.96), accuracy of 0.87 (95% CI, 0.84-0.89), sensitivity of 0.90 (95% CI, 0.88-0.92), specificity of 0.84 (95% CI, 0.82-0.86), PPV of 0.84 (95% CI, 0.83-0.86), and NPV of 0.90 (95% CI, 0.89-0.92) (Supplementary Fig 11, online only).

## Discussion

We used data from a large clinical registry (VQI) consisting of 33,924 patients who underwent varicose vein ablation for CEAP C2 to C4 disease to develop ML models that accurately predict 1-year postprocedural LCI with AUROCs of >0.90. There were several key findings. First, patients who do not experience clinical improvement after varicose vein ablation have predictive features at the preoperative, intraoperative, and postoperative stages. ML-based modeling allowed us to assess the combined impact of these factors on LCI risk. Second, we evaluated six ML models on our dataset and XGBoost achieved the best performance. The XGBoost algorithm demonstrated excellent discrimination and calibration across the preoperative, intraoperative, and postoperative stages. Furthermore, predictive performance remained robust across demographic and clinical subpopulations. Third, although intraoperative and postoperative factors contributed to long-term risk, most of the top 10 predictors for 1-year LCI were preoperative features. This finding highlights an important opportunity for our risk prediction tool to guide patient selection and preoperative care. These models have the potential to support clinical decision-making throughout a patient's perioperative course, thereby facilitating individualized risk assessment, counselling, and management.

### Comparison with the existing literature

Clinical outcomes in patients undergoing varicose vein ablation have been characterized previously, but there are no existing validated models to predict the risk of LCI.^9^ Rodríguez et al^9^ recently assessed LCI after varicose vein ablation using VQI data from 2014 to 2023. They demonstrated that 16.1% of patients experienced LCI after varicose vein ablation based on the same definition of preoperative VCSS minus postoperative VCSS in our study.^9^ We demonstrated a similar LCI rate of 16.6% in our updated 2014-2024 VQI cohort. Rodríguez et al^9^ showed that older age, Black race, and lower severity of disease were associated with LCI. We similarly demonstrated that these variables were important predictors of LCI. Notably, the top three predictors for LCI in our ML models were VCSS, VVSymQ score, CEAP classification. Specifically, patients with lower VCSS, VVSymQ scores, and CEAP classifications, indicating lower severity of disease, were less likely to experience clinical improvement after vein ablation. Rodríguez et al^9^ also showed that the incidence of complications was low, with no differences between patients with vs without LCI. We showed similar findings, with an overall complication rate of <0.3% in our cohort, highlighting that varicose vein ablation is a relative safe procedure. Similar to Rodríguez et al,^9^ we showed that patients with LCI were less likely to routinely use compression therapy before intervention, highlighting a potential role for optimizing compression therapy in patients at high risk for LCI. There were two key differences between our study and the paper by Rodríguez et al.^9^ First, whereas Rodríguez et al^9^ used VQI data up to 2023, we included a slightly more contemporary cohort up to 2024. Second, and more important, whereas Rodríguez et al^9^ assessed factors associated with LCI after vein ablation using multivariable logistic regression, our work focused on building highly accurate prediction models for 1-year LCI using advanced ML techniques. Overall, we built on the work of Rodríguez et al^9^ by not only characterizing the incidence of LCI and assessing factors associated with LCI, but also developing ML-based predictive models for 1-year LCI after varicose vein ablation, which may provide greater utility in the routine clinical setting to guide decision-making.

Bonde et al^18^ trained ML algorithms on a cohort of patients who underwent >2900 unique procedures in the NSQIP database to predict perioperative complications, achieving AUROC's between 0.85 and 0.88. Given that patients being considered for varicose vein ablation are generally a unique population characterized by specific age ranges, comorbidities, and clinical presentations, the usefulness of generic risk prediction tools may have limitations.^54^ By developing ML algorithms tailored to patients undergoing varicose vein ablation, we achieved an AUROC of >0.90. Furthermore, our model has been trained to predict 1-year LCI, which is of clinical importance to vascular surgeons and interventionalists.^55^ Therefore, we demonstrate the value of building procedure-specific ML models, which can improve performance and clinical applicability. This prediction model for varicose vein ablation complements our previously described ML algorithms for predicting outcomes after arterial interventions, which achieved similarly excellent performance with AUROCs of ≥0.90.^20^, ^21^, ^22^, ^23^, ^24^, ^25^

### Explanation of findings

There are several explanations for our findings. First, patients who experience LCI after varicose vein ablation represent a unique population with multiple risk factors, such as lower preoperative VCSS, VVSymQ scores, and CEAP classifications, which is corroborated by the previous literature.^9^ This result could be explained by the fact that these patients may have less severe venous reflux, and, therefore, vein ablation may not improve their symptoms significantly.^9^ Given that most components of the VCSS reflect patient symptoms rather than cosmetics, the lack of improvement in symptoms after vein ablation was likely an important contributor to LCI in this study. We also demonstrated that patients with prior varicose vein ablations were more likely to experience LCI. This finding aligns with the prior literature and suggests that recurrent venous reflux disease may be more challenging to manage.^56^^,^^57^ Additionally, we demonstrated that patients undergoing open surgical treatment were more likely to experience 1-year LCI than patients undergoing endovenous ablation. This finding corroborates the SVS, American Venous Forum, and American Vein, and Lymphatic Society guidelines recommending endovenous over surgical treatment as first-line therapy in patients with symptomatic varicose veins.^6^^,^^7^ Furthermore, we showed that patients with 1-year LCI were less likely to use compression stockings routinely preoperatively. This finding highlights a potential avenue to optimize lifestyle management of patients with chronic venous disease before offering intervention.^58^ Another interpretation could be that patients who do not use compression stockings routinely preoperatively are less likely to have their symptoms relieved by compression therapy if the symptoms are not related to venous insufficiency. In this cohort, vein ablation is likely less effective. Therefore, a patient's preoperative use of and response to compression therapy may be an important decision-making point to guide clinicians in terms of whether to offer vein ablation.^59^

Second, we demonstrated that preoperative features can accurately predict 1-year LCI after varicose vein ablation. This finding suggests that the ability to predict 1-year LCI accurately based on VCSS scores after varicose vein ablation can be established preprocedurally to support decision-making regarding patient selection, counselling, periprocedural management, and follow-up. The fact that the predictive features align with clinical intuition regarding risk factors for LCI after vein ablation highlight the explainability and potential clinical utility of the ML algorithms.^6^, ^7^

Third, our ML models performed better than existing tools for several potential reasons. Compared with traditional logistic regression, advanced ML techniques can better model complex, nonlinear relationships between inputs and outputs.^60^ This factor is especially important in health care data, because patient outcomes can be influenced by many demographic, clinical, and system-level factors.^61^ Our top-performing algorithm was XGBoost, which has unique advantages, including relatively fewer issues with overfitting and faster computing while maintaining precision.^62^, ^63^, ^64^ Furthermore, XGBoost works well with structured data, which may explain its superior performance compared with more complex algorithms, such as neural networks on our dataset.^65^

Fourth, the performance of our models remained robust on subgroup analyses of specific demographic and clinical populations. This finding is important, given that algorithm bias against underrepresented populations is a frequently encountered issue in ML models.^66^ We were likely able to avoid such biases owing to the excellent capture of sociodemographic data by VQI.^19^ Specific to this study, females have a higher incidence of chronic venous disease compared with males.^2^ Given that ML models have been reported to underperform in females owing to biases in the data used to train the models,^67^ it was important that we demonstrated excellent model performance in both females and males.

## Implications

The programming code used to develop our ML models is publicly available on GitHub, allowing clinicians involved in the perioperative management of patients being considered for varicose vein treatment to use our tool. At a system-wide level, our models can be implemented by the >1000 VQI participating centers.^19^ The VQI database managers at these institutions routinely capture the input features used in our ML algorithms.^19^ The number of VQI centers has grown considerably from 400 in 2019 to >1000 in 2023.^19^^,^^68^ Recently, the VQI recorded >1 million procedures.^69^ Therefore, our models have broad and growing usefulness. They also have potential for use beyond VQI sites, as our predictors are commonly captured variables for the routine care of patients with chronic venous disease.^70^ Given the challenges of deploying prediction models into practice, thoughtful consideration of implementation science principles is critical.^71^ A key advantage of our ML models is their ability to provide automated risk predictions, thereby enhancing feasibility in busy clinical settings compared with traditional risk predictors that often require manual input of variables.^13^ Specifically, our ML algorithms can autonomously extract a patient's VQI data to generate risk predictions. Given the goal of automation, whereby our model can automatically extract VQI data to make risk predictions, there is no significant value to reducing the number of input features with the trade-off of a potential reduction in predictive performance. Therefore, a simplified model with a reduced number of input features was not created. As such, a nomogram was not developed as this would require a simplified model with fewer input features, manual calculations of predicted risk, and potentially reduced model performance. To use our model on a given patient, it would be important to apply our programming code to extract that patient's VQI data to automatically generate a risk prediction using all available features. To facilitate successful implementation of our ML tool, we recommend establishing and supporting data analytics teams at the institutional level, who can integrate our source code into clinical workflows. Such teams can provide important benefits to patient care, and their expertise can facilitate the deployment of our ML models.^72^

## Limitations

Our study has several limitations. First, our models were developed with VQI data, a voluntary registry primarily comprising data from North American centers. Future studies are needed to assess whether performance can be generalized beyond VQI sites. Notably, there is a critical need for external validation of prediction models on independent datasets to assess their generalizability, as demonstrated by Steyerberg and Harrell.^73^ Specifically, good internal performance does not guarantee external validity.^73^ Importantly, there are demographic and health care system differences between North America and other regions in the world, which may limit the generalizability of our VQI-based algorithm. It would, therefore, be prudent to validate the model on non-North American cohorts in future studies. Furthermore, although the creation of a testing cohort that was not used for model training decreased the risk of overfitting, external validation on independent datasets would further mitigate this concern. Additionally, only patients with CEAP class C2 to C4 disease were included in this study. Development of future predictive models for class C5 or C6 disease may further expand clinical guidance for patients with more severe venous disease. Second, although we evaluated six different ML models, there are other ML models available. We chose these six models because of their established efficacy for predicting postoperative outcomes.^32^ We achieved excellent performance; however ongoing evaluation of novel ML techniques would be prudent. For example, other deep learning models and ensemble methods such as stacking or bagging techniques were not evaluated owing to their complex architecture and potential for reduced explainability.^74^^,^^75^ Given that model explainability is important in the health care setting to build trust in clinicians to use the algorithms routinely, we opted to test less complex and more explainable models.^76^ As complex algorithms become more explainable, it would be prudent to train and evaluate these models in the future for potentially improved predictive performance. Third, vein ablation technical success was not captured in our dataset; therefore, we could not assess the correlation between technical success and 1-year LCI. Fourth, our definition of LCI was based on preoperative and postoperative VCSS scores, a physician-reported measure.^31^ The VVSymQ is a patient-reported measure that may be more representative of patients' experience of disease.^77^ Although preoperative VVSymQ scores were reported, 1-year postoperative VVSymQ scores were not well-captured in the VQI database. Therefore, changes in VVSymQ scores could not be calculated reliably and were not included in the outcome of interest to be predicted by the ML models. Additionally, although the VCSS and VVSymQ capture symptoms of venous disease, they do not evaluate comprehensively activities of daily living, self-reported quality of life, or psychological/social dependency factors captured in other scores such as the Chronic Venous Disease Quality of Life Questionnaire^78^ and Venous Insufficiency Epidemiological and Economic Study Questionnaire,^79^ which are not recorded by VQI. Future predictive models trained to predict more comprehensive patient-reported outcome measures for varicose vein disease severity using updated datasets may provide further utility to guide patient-centered clinical decision-making.

## Conclusions

We used a large, vascular-specific clinical registry (VQI) to develop robust ML models that predict 1-year LCI after varicose vein ablation for CEAP class C2 to C4 disease with excellent performance (AUROCs of >0.90). Our models can be applied across the preoperative, intraoperative, and postoperative stages to guide patient counselling and clinical decision-making regarding strategies to mitigate the risk of LCI. Notably, our models remained robust across demographic and clinical subpopulations and outperformed existing prediction tools and logistic regression, and therefore, have potential for important utility in the care of patients with chronic venous disease. Prospective validation of our ML algorithms is warranted.

## Author Contributions

Conception and design: BL, NE, DB, CM, MM, GR, MA

Analysis and interpretation: BL, NE, DB, DL, LA, DW, MH, OR, CM, MM, GR, MA

Data collection: BL, NE

Writing the article: BL

Critical revision of the article: BL, NE, DB, DL, LA, DW, MH, OR, CM, MM, GR, MA

Final approval of the article: BL, NE, DB, DL, LA, DW, MH, OR, CM, MM, GR, MA

Statistical analysis: BL, DB

Obtained funding: BL

Overall responsibility: MA

## Funding

This research was partially funded by the 10.13039/501100000024Canadian Institutes of Health Research, Ontario Ministry of Health, 10.13039/501100000241PSI Foundation, and Schwartz Reisman Institute for Technology and Society at the University of Toronto (to B.L.). The funding sources did not play a role in study design, collection, analysis, or interpretation of data, manuscript writing, creation of the manuscript, or the decision to submit the manuscript for publication.

## Disclosures

None.
