# Supplementary material for: Predicting lack of clinical improvement following varicose vein ablation using machine learning
Source: J Vasc Surg Venous Lymphat Disord. 2024 Dec 26;13(3):102162. doi: 10.1016/j.jvsv.2024.102162 (PMC11803835; doi:10.1016/j.jvsv.2024.102162)
Supplement: Supplementary Tables and Figures (online only) [file mmc1.docx]

**Supplementary Table 1. Pre-operative features for machine learning models**

| **Features (n = 111)** | **Definition based on Vascular Quality Initiative Data Dictionary** |
| --- | --- |
| **Logistics** |  |
| Region | Region of procedure (deidentified) |
| Center | Center of procedure (deidentified) |
| Physician | Physician who performed procedure (deidentified) |
| Intervention year | Year of intervention |
| Intervention month | Month of intervention |
| Intervention weekday | Day of week that intervention was performed |
| **Demographics** |  |
| Age | Age in years |
| Sex | Male or female |
| Body mass index | Weight in kg / height in m^2^ |
| Race | Patient-reported race including White, Black, Asian, American Indian or Alaskan Native, Native Hawaiian or other Pacific Islander, more than 1 race, or unknown/other |
| Ethnicity | Hispanic or non-Hispanic |
| Primary insurer | Primary method of health insurance including Medicare, Medicaid, Commercial, Medicare Advantage, Military or Veterans Affairs, Non-US Insurance, self-pay (uninsured), or unknown/other |
| Rural residence | Defined based on the patient’s primary rural-urban commuting area (RUCA) code based on the most recent publicly available dataset. Rural residence is RUCA code 10 (<https://www.ers.usda.gov/data-products/rural-urban-commuting-area-codes/documentation//>). |
| Median Area Deprivation Index (ADI) | National percentile rank based on the most recent publicly available dataset. Calculated by taking the median ADI national percentile rank among all 9-digit zip code records that contain the patient’s 5-digit zip code prefix (<https://www.neighborhoodatlas.medicine.wisc.edu/>). A higher number indicates a greater level of socioeconomic disadvantage, accounting for factors such as income, education, employment, and housing quality. |
| **Medical history** |  |
| Prior deep vein thrombosis | Clinical history of prior deep vein thrombosis based on documentation in the medical record confirmed by imaging reported as 1) ipsilateral, 2) contralateral, 3) bilateral, or 4) side not reported |
| Prior superficial phlebitis | Clinical history of prior lower extremity superficial phlebitis reported as 1) ipsilateral, 2) contralateral, 3) bilateral, or 4) side not reported |
| Prior pulmonary embolism | Clinical history of pulmonary embolism based on documentation in medical record confirmed by imaging |
| Prior varicose vein ablation | Prior endovascular or surgical varicose vein treatment |
| Prior thermal radiofrequency ablation on right leg | Prior ablation using radiofrequency whereby bipolar currents cause heat damage to the treated vein on the right leg |
| Prior thermal laser ablation on right leg | Prior ablation using any laser that heats the vein causing steam damage to the treated vein on the right leg |
| Prior mechanochemical ablation on right leg | Prior ablation using a sclerosing agent along with a wire that vibrates simultaneously to cause damage to the treated vein on the right leg |
| Prior chemical ablation on right leg | Prior sclerosing agent that was injected in the vein that causes chemical irritation and damage to the treated vein on the right leg |
| Prior embolic adhesive ablation on right leg | Prior glue injected into the vessel that causes the vein to close on the right leg |
| Prior surgical treatment for varicose veins on right leg | Prior stripping or excision of truncal veins or vein clusters or ligation of perforating veins on the right leg |
| Prior high ligation and stripping on right leg | Prior ligation at vein insertion, including saphenofemoral or saphenopopliteal junctions, combined with stripping of the associated vein on the right leg |
| Prior vein stripping on right leg | Prior removal of veins via a surgical incision and a catheter placed through the vein to allow it to be removed over a long distance through 1-2 smaller incisions on the right leg |
| Prior stab phlebectomy on right leg | Prior removal of veins via small incisions, including use of hooks, on the right leg |
| Prior trivex phlebectomy on right leg | Prior trans illuminated powered phelectomy on the right leg |
| Prior open ligation of vein on right leg | Prior surgical ligation of veins through an incision on the right leg |
| Prior endoscopic vein ligation on right leg | Prior ligation of a vein using an endoscopic device on the right leg |
| Prior thermal radiofrequency ablation on left leg | Prior ablation using radiofrequency whereby bipolar currents cause heat damage to the treated vein on the left leg |
| Prior thermal laser ablation on left leg | Prior ablation using any laser that heats the vein causing steam damage to the treated vein on the left leg |
| Prior mechanochemical ablation on left leg | Prior ablation using a sclerosing agent along with a wire that vibrates simultaneously to cause damage to the treated vein on the left leg |
| Prior chemical ablation on left leg | Prior sclerosing agent that was injected in the vein that causes chemical irritation and damage to the treated vein on the left leg |
| Prior embolic adhesive ablation on left leg | Prior glue injected into the vessel that causes the vein to close on the left leg |
| Prior surgical treatment for varicose veins on left leg | Prior stripping or excision of truncal veins or vein clusters or ligation of perforating veins on the left leg |
| Prior high ligation and stripping on left leg | Prior ligation at vein insertion, including saphenofemoral or saphenopopliteal junctions, combined with stripping of the associated vein on the left leg |
| Prior vein stripping on left leg | Prior removal of veins via a surgical incision and a catheter placed through the vein to allow it to be removed over a long distance through 1-2 smaller incisions on the left leg |
| Prior stab phlebectomy on left leg | Prior removal of veins via small incisions, including use of hooks, on the left leg |
| Prior trivex phlebectomy on left leg | Prior trans illuminated powered phelectomy on the left leg |
| Prior open ligation of vein on left leg | Prior surgical ligation of veins through an incision on the left leg |
| Prior endoscopic vein ligation on left leg | Prior ligation of a vein using an endoscopic device on the left leg |
| Number of pregnancies | Number of full term or incomplete pregnancies |
| Pre-operative anticoagulation | Any anticoagulant taken prior to the procedure |
| Reason for pre-operative anticoagulation | Reason for taking pre-operative anticoagulation including 1) prophylactic prevention of venous thromboembolism, 2) therapeutic treatment of venous thromboembolism, 3) arrhythmia, or 4) other reason |
| Anticoagulant type | Type of anticoagulant taken including 1) vitamin K antagonist, 2) dabigatran, 3) rivaroxaban, 4) unfractionated heparin, 5) low molecular weight heparin, 6) apixaban, 7) edoxaban, 8) betrixaban, 9) fondaparinux, 10) enoxaparin sodium, or 11) other |
| **Clinical presentation** |  |
| Side treated | Leg to be treated including 1) right, 2) left, or 3) bilateral |
| CEAP classification | CEAP classification of treated leg including:  C1: Telangiectasias or reticular veins;  C2: Varicose veins;  C2r: Recurrent Varicose veins;  C3: Edema;  C4a: Pigmentation and eczema;  C4b: Lipodermatosclerosis and atrophie blanche;  C4c: Corona phlebectatica |
| Venous clinical severity score | Revised venous clinical severity score that assesses the clinical severity of venous insufficiency, scored out of 30 based on 10 components below with a maximum score of 3 each, higher score means greater severity |
| Pain | Pain or other discomfort, i.e., aching, heaviness, fatigue, soreness, burning.  None (0) = No pain,  Mild (1) = Occasional pain or other discomfort, not restricting regular daily activity,  Moderate (2) = Moderate daily pain or other discomfort interfering with but not preventing regular daily activities,  Severe (3) = Daily pain or discomfort that limits most regular daily activities. |
| Varicose veins | Varicose veins defined as greater than or equal to 3mm in diameter:  None (0) = No qualifying varicose veins,  Mild (1) = Few scattered isolated branch varicosities or clusters; also includes corona phlebectatica (ankle flare) defined as > 5 blue telangiectasia at the inner or outer edge of the foot;  Moderate (2) = Multiple varicose veins that are confined to calf or thigh;  Severe (3) = Multiple varicose veins that involve both the calf and thigh |
| Venous edema | Edema of venous origin:  None (0) = No edema,  Mild (1) = Limited to foot and ankle area,  Moderate (2) = Extends above ankle but below knee,  Severe (3) = Extends to knee and above. |
| Skin pigmentation | Edema of venous origin; does not include focal pigmentation over varicose veins or pigmentation due to other chronic disease (i.e., vasculitis purpura)  None (0) = No skin pigmentation or focal pigmentation that is confined to the skin over the varicose veins,  Mild (1) = Pigmentation that is limited to the perimalleolar area,  Moderate (2) = Diffuse pigmentation that involves the lower third of the calf,  Severe (3) = Diffuse pigmentation that involves more than the lower third of the calf. |
| Inflammation | Inflammation that is more than just recent pigmentation (i.e., erythema, cellulitis, venous eczema, dermatitis)  None (0) = No inflammation as defined,  Mild (1) = Inflammation that is limited to the perimalleolar area,  Moderate (2) = Inflammation that involves the lower third of the calf,  Severe (3) = Inflammation that involves more than the lower third of the calf. |
| Induration | Induration of venous origin of secondary skin and subcutaneous changes (i.e., chronic edema with fibrosis, hypodermitis), including white atrophy and lipodermatosclerosis  None (0) = No induration as defined,  Mild (1) = Induration that is limited to the perimalleolar area,  Moderate (2) = Induration that involves the lower third of the calf,  Severe (3) = Induration that involves more than the lower third of the calf. |
| Active ulcer number | Number of discrete non-healed venous ulcers.  0 = 0 ulcers  1 = 1 ulcer  2 = 2 ulcers  3 = greater than or equal to 3 ulcers |
| Active ulcer duration | Duration of most chronic ulcer  0 = none  1 = < 3 months  2 = 3-12 months  3 = > 12 months |
| Active ulcer size | Maximum diameter of largest venous ulcer; if not round, record longest elliptical axis  0 = none  1 = < 2 cm  2 = 2-6 cm  3 = > 6 cm |
| Use of compression therapy | Current usage of compression treatment therapy (stockings, bandage, etc.) by patient history.  No (0) = No compression therapy used;  Intermittent (1) = Compression therapy applied intermittently;  Most days (2) = Compression therapy applied almost every day with a few days skipped once and awhile;  Every day (3) = Compression therapy used every day. |
| Number of healed ulcers | Number of healed venous ulcers by history or healed scar: 0 = 0 ulcers, 1 = 1 ulcer, 2 = 2 ulcers, 3 = greater than or equal to 3 ulcers |
| Varicose vein symptom questionnaire (VVSymQ) score | A patient-reported outcome measure of varicose vein symptoms based on 5 components below with a score of 5 each for a total score of 25, higher score means more symptomatic and/or greater impact on patients; patient is asked “Since waking up today, how often had you had the following problem in your leg to be treated?” This question was asked for each of the following five symptoms: heaviness, achiness, swelling, throbbing, and itching. |
| Heaviness | Patient feels heaviness in their leg from varicose veins: 0 = None of the time, 1 = A little of the time, 2 = Some of the time, 3 = A good bit of the time, 4 = Most of the time, 5 = All of the time |
| Achiness | Patient feels achiness in their leg from varicose veins: 0 = None of the time, 1 = A little of the time, 2 = Some of the time, 3 = A good bit of the time, 4 = Most of the time, 5 = All of the time |
| Swelling | Patient feels swelling in their leg from varicose veins: 0 = None of the time, 1 = A little of the time, 2 = Some of the time, 3 = A good bit of the time, 4 = Most of the time, 5 = All of the time |
| Throbbing | Patient feels throbbing in their leg from varicose veins: 0 = None of the time, 1 = A little of the time, 2 = Some of the time, 3 = A good bit of the time, 4 = Most of the time, 5 = All of the time |
| Itching | Patient feels itching in their leg from varicose veins: 0 = None of the time, 1 = A little of the time, 2 = Some of the time, 3 = A good bit of the time, 4 = Most of the time, 5 = All of the time |
| Appearance | Patient-reported appearance of varicose veins: 0 = Not at all noticeable, 1 = Slightly noticeable, 2 = Moderately noticeable, 3 = Very noticeable, 4 = Extremely noticeable |
| Work impact | Patient-reported impact of varicose veins on their ability to work or perform daily activities: 0 = None, 1 = Symptoms present but full work/activity, 2 = Mildly reduced work/activity, 3 = Moderately reduced work/activity, 4 = Severely reduced work/activity, 5 = Unable to do work/activity |
| **Anatomic and physiologic characteristics** |  |
| Imaging | Type of pre-operative imaging of superficial veins regarding reflux including 1) none, 2) duplex ultrasound, or 3) other (e.g., computed tomography venography, magnetic resonance venography, conventional venography) |
| Reflux on duplex ultrasound > 0.5 seconds |  |
| None | No reflux seen in any veins in treated leg |
| GSV thigh | Reflux > 0.5 seconds seen in GSV in the thigh in the treated leg |
| GSV calf | Reflux > 0.5 seconds seen in GSV in the calf in the treated leg |
| Anterior accessory GSV thigh | Reflux > 0.5 seconds seen in anterior accessory GSV in the thigh in the treated leg |
| Anterior accessory GSV calf | Reflux > 0.5 seconds seen in anterior accessor GSV in the calf in the treated leg |
| Superficial accessory GSV | Reflux > 0.5 seconds seen in superficial accessory GSV in the treated leg |
| GSV remnant | Reflux > 0.5 seconds seen in segment of residual GSV after previous treatment in the treated leg |
| Small saphenous vein | Reflux > 0.5 seconds seen in small saphenous vein in the treated leg |
| Deep veins | Reflux > 0.5 seconds seen in deep veins in the treated leg |
| Other veins | Reflux > 0.5 seconds seen in other veins in the treated leg, including the posterior accessory great saphenous vein, anterior thigh perforating vein, posterior thigh circumflex vein, intersaphenous veins including the Giacomini vein, lateral thigh veins, perforating veins in the thigh, perforating veins at the knee, perforating veins in the calf, or perforating veins at the ankle |
| Not studied | Reflux on duplex ultrasound not studied in the treated leg |
| Thrombus on duplex ultrasound |  |
| None | No thrombus seen in any veins of treated leg |
| GSV thigh | Thrombus seen in GSV in the thigh in the treated leg |
| GSV calf | Thrombus seen in GSV in the calf in the treated leg |
| Anterior accessory GSV thigh | Thrombus seen in anterior accessory GSV in the thigh in the treated leg |
| Anterior accessory GSV calf | Thrombus seen in anterior accessor GSV in the calf in the treated leg |
| Superficial accessory GSV | Thrombus seen in superficial accessory GSV in the treated leg |
| GSV remnant | Thrombus seen in segment of residual GSV after previous treatment in the treated leg |
| Small saphenous vein | Thrombus seen in small saphenous vein in the treated leg |
| Deep veins | Thrombus seen in deep veins in the treated leg |
| Other veins | Thrombus seen in other veins in the treated leg, including the posterior accessory great saphenous vein, anterior thigh perforating vein, posterior thigh circumflex vein, intersaphenous veins including the Giacomini vein, lateral thigh veins, perforating veins in the thigh, perforating veins at the knee, perforating veins in the calf, or perforating veins at the ankle |
| Not studied | Thrombus on duplex ultrasound not studied in the treated leg |
| Reflux in additional veins seen on duplex ultrasound > 0.5 seconds |  |
| Posterior accessory GSV thigh | Reflux > 0.5 seconds seen in the posterior accessory GSV in the thigh in the treated leg |
| Posterior accessory GSV calf | Reflux > 0.5 seconds seen in the posterior accessory GSV in the calf in the treated leg |
| Anterior thigh perforating vein | Reflux > 0.5 seconds seen in the anterior thigh perforating vein in the treated leg |
| Posterior thigh circumflex vein | Reflux > 0.5 seconds seen in the posterior thigh circumflex vein in the treated leg |
| Intersaphenous veins | Reflux > 0.5 seconds seen in the intersaphenous veins, including branches between the GSV and small saphenous vein including the Giacomini vein, in the treated leg |
| Lateral thigh veins | Reflux > 0.5 seconds seen in the lateral thigh veins from the hip to the knee in the treated leg |
| Perforating veins in thigh | Reflux > 0.5 seconds seen in the perforating veins in the thigh in the treated leg |
| Perforating veins at knee | Reflux > 0.5 seconds seen in the perforating veins at the knee in the treated leg |
| Perforating veins in calf | Reflux > 0.5 seconds seen in the perforating veins in the calf in the treated leg |
| Perforating veins at ankle | Reflux > 0.5 seconds seen in the perforating veins at the ankle in the treated leg |
| Thrombus in additional veins on duplex ultrasound |  |
| Posterior accessory GSV thigh | Thrombus seen in the posterior accessory GSV in the thigh in the treated leg |
| Posterior accessory GSV calf | Thrombus seen in the posterior accessory GSV in the calf in the treated leg |
| Anterior thigh perforating vein | Thrombus seen in the anterior thigh perforating vein in the treated leg |
| Posterior thigh circumflex vein | Thrombus seen in the posterior thigh circumflex vein in the treated leg |
| Intersaphenous veins | Thrombus seen in the intersaphenous veins, including branches between the GSV and small saphenous vein including the Giacomini vein, in the treated leg |
| Lateral thigh veins | Thrombus seen in the lateral thigh veins from the hip to the knee in the treated leg |
| Perforating veins in thigh | Thrombus seen in the perforating veins in the thigh in the treated leg |
| Perforating veins at knee | Thrombus seen in the perforating veins at the knee in the treated leg |
| Perforating veins in calf | Thrombus seen in the perforating veins in the calf in the treated leg |
| Perforating veins at ankle | Thrombus seen in the perforating veins at the ankle in the treated leg |

Abbreviations: CEAP (Clinical-Etiological-Anatomical-Pathophysiological), GSV (great saphenous vein).

**Supplementary Table 2. Intra-operative features for machine learning models**

| **Features (n = 100)** | **Definition based on Vascular Quality Initiative Data Dictionary** |
| --- | --- |
| Anesthesia |  |
| None | No anesthesia used |
| Minimal sedation | A drug-induced state during which the patient responds normally to verbal commands. Cognitive function and coordination may be impaired. Ventilatory and cardiovascular functions are unaffected. |
| Moderate sedation | A drug-induced depression of consciousness during which the patient responds purposefully to verbal command, either alone or accompanied by light tactile stimulation. No interventions are necessary to maintain a patent airway. Spontaneous ventilation is adequate. |
| Deep sedation | A drug-induced depression of consciousness during which the patient cannot be easily aroused but responds purposefully following repeated or painful stimulation. Independent ventilatory function may be impaired. The patient may require assistance to maintain a patent airway. Spontaneous ventilation may be inadequate. |
| Local | Locally injected anesthesia at incision or puncture site. |
| Tumescent | Infusion of local anesthetic into a region, usually with an infusion pump over a large region. |
| Regional | Any nerve block, spinal, or epidural. |
| General | Drug-induced loss of consciousness during which patients are not arousable, even by painful stimulation. The ability to independently maintain ventilatory function is often impaired. Patients often require assistance in maintaining a patent airway, and positive pressure ventilation may be required because of depressed spontaneous ventilation or drug-induced depression of neuromuscular function. Cardiovascular function may be impaired. |
| Peri-procedural anticoagulation | Anticoagulation given just before and/or immediately after the procedure including 1) low molecular weight heparin, 2) unfractionated heparin, or 3) other anticoagulant |
| Number of veins treated | Total number of veins treated during procedure |
| Side of primary vein treated | Right or left leg |
| Side of second vein treated if applicable | Right or left leg |
| Side of third vein treated if applicable | Right or left leg |
| Side of fourth vein treated if applicable | Right or left leg |
| Side of fifth vein treated if applicable | Right or left leg |
| Side of sixth vein treated if applicable | Right or left leg |
| Location of primary vein treated | Truncal = Major axial superficial vein in thigh or leg. These includes the great saphenous vein; superficial accessory great saphenous vein; anterior accessory great saphenous vein; small saphenous vein.  Truncal recanalyzed/remnant = A truncal vein that was previously treated but remain patent in total or in a clinically significant remnant;  Cluster = Superficial varicosities;  Perforator = Connecting vein between superficial and deep system;  Perforator recanalyzed = Previously treated perforating vein that remains patent. |
| Location of second vein treated if applicable | Same as above for second vein treated if applicable |
| Location of third vein treated if applicable | Same as above for third vein treated if applicable |
| Location of fourth vein treated if applicable | Same as above for fourth vein treated if applicable |
| Location of fifth vein treated if applicable | Same as above for fifth vein treated if applicable |
| Location of sixth vein treated if applicable | Same as above for sixth vein treated if applicable |
| Specific truncal vein treated for primary vein treated if applicable | 0 = GSV thigh and calf, 1 = GSV thigh, 2 = GSV calf, 3 = superficial accessory GSV thigh, 4 = anterior accessory GSV thigh, 5 = anterior accessory GSV calf, 6 = small saphenous vein thigh extension, 7 = small saphenous vein calf, 8 = other truncal vein |
| Specific truncal vein treated for second vein treated if applicable | Same as above for second vein treated if applicable |
| Specific truncal vein treated for third vein treated if applicable | Same as above for third vein treated if applicable |
| Specific truncal vein treated for fourth vein treated if applicable | Same as above for fourth vein treated if applicable |
| Specific truncal vein treated for fifth vein treated if applicable | Same as above for fifth vein treated if applicable |
| Specific truncal vein treated for sixth vein treated if applicable | Same as above for sixth vein treated if applicable |
| Specific perforator veins treated for primary vein treated if applicable | 1) Thigh perforators; includes perforator of Dodd and Hatch.  2) Calf perforators; includes perforators of Boyd, Cockett, Sherman.  3) Ankle perforators; includes foot perforators. |
| Specific perforator veins treated for second vein treated if applicable | Same as above for second vein treated if applicable |
| Specific perforator veins treated for third vein treated if applicable | Same as above for third vein treated if applicable |
| Specific perforator veins treated for fourth vein treated if applicable | Same as above for fourth vein treated if applicable |
| Specific perforator veins treated for fifth vein treated if applicable | Same as above for fifth vein treated if applicable |
| Specific perforator veins treated for sixth vein treated if applicable | Same as above for sixth vein treated if applicable |
| Specific other truncal vein treated for primary vein treated if applicable | 1 = posterior accessory GSV thigh, 2 = posterior accessory GSV calf, 3 = anterior thigh circumflex vein, 4 = posterior thigh circumflex vein, 5 = intersaphenous veins, 6 = superficial veins of the lateral thigh, 7 = Saphenofemoral junction, 8 = Saphenopopliteal junction, 9 = Other |
| Specific other truncal vein treated for second vein treated if applicable | Same as above for second vein treated if applicable |
| Specific other truncal vein treated for third vein treated if applicable | Same as above for third vein treated if applicable |
| Specific other truncal vein treated for fourth vein treated if applicable | Same as above for fourth vein treated if applicable |
| Specific other truncal vein treated for fifth vein treated if applicable | Same as above for fifth vein treated if applicable |
| Specific other truncal vein treated for sixth vein treated if applicable | Same as above for sixth vein treated if applicable |
| Maximum diameter of primary vein treated | Largest diameter in mm, including any varices within the vein segment |
| Maximum diameter of second vein treated if applicable | Same as above for second vein treated if applicable |
| Maximum diameter of third vein treated if applicable | Same as above for third vein treated if applicable |
| Maximum diameter of fourth vein treated if applicable | Same as above for fourth vein treated if applicable |
| Maximum diameter of fifth vein treated if applicable | Same as above for fifth vein treated if applicable |
| Maximum diameter of sixth vein treated if applicable | Same as above for sixth vein treated if applicable |
| Treatment type of primary vein treated | 1 = Thermal radiofrequency ablation, 2 = Thermal laser ablation, 3 = Surgery, 4 = Other endovenous treatment |
| Treatment type of second vein treated if applicable | Same as above for second vein treated if applicable |
| Treatment type of third vein treated if applicable | Same as above for third vein treated if applicable |
| Treatment type of fourth vein treated if applicable | Same as above for fourth vein treated if applicable |
| Treatment type of fifth vein treated if applicable | Same as above for fifth vein treated if applicable |
| Treatment type of sixth vein treated if applicable | Same as above for sixth vein treated if applicable |
| Length of primary vein treated | Measured vein length in cm |
| Length of second vein treated if applicable | Same as above for second vein treated if applicable |
| Length of third vein treated if applicable | Same as above for third vein treated if applicable |
| Length of fourth vein treated if applicable | Same as above for fourth vein treated if applicable |
| Length of fifth vein treated if applicable | Same as above for fifth vein treated if applicable |
| Length of sixth vein treated if applicable | Same as above for sixth vein treated if applicable |
| Tip length for radiofrequency ablation of primary vein treated if applicable | 2.5cm, 3cm, 7cm, 10cm, radiofrequency stylet (perforator), or other |
| Tip length for radiofrequency ablation of second vein treated if applicable | Same as above for second vein treated if applicable |
| Tip length for radiofrequency ablation of third vein treated if applicable | Same as above for third vein treated if applicable |
| Tip length for radiofrequency ablation of fourth vein treated if applicable | Same as above for fourth vein treated if applicable |
| Tip length for radiofrequency ablation of fifth vein treated if applicable | Same as above for fifth vein treated if applicable |
| Tip length for radiofrequency ablation of sixth vein treated if applicable | Same as above for sixth vein treated if applicable |
| Watts for laser ablation of primary vein treated if applicable | Power setting in watts for laser ablation if applicable |
| Watts for laser ablation of second vein treated if applicable | Same as above for second vein treated if applicable |
| Watts for laser ablation of third vein treated if applicable | Same as above for third vein treated if applicable |
| Watts for laser ablation of fourth vein treated if applicable | Same as above for fourth vein treated if applicable |
| Watts for laser ablation of fifth vein treated if applicable | Same as above for fifth vein treated if applicable |
| Watts for laser ablation of sixth vein treated if applicable | Same as above for sixth vein treated if applicable |
| Foam sclerotherapy gas for primary vein treated if applicable | Air, carbon dioxide, or other |
| Foam sclerotherapy gas for second vein treated if applicable | Same as above for second vein treated if applicable |
| Foam sclerotherapy gas for third vein treated if applicable | Same as above for third vein treated if applicable |
| Foam sclerotherapy gas for fourth vein treated if applicable | Same as above for fourth vein treated if applicable |
| Foam sclerotherapy gas for fifth vein treated if applicable | Same as above for fifth vein treated if applicable |
| Foam sclerotherapy gas for sixth vein treated if applicable | Same as above for sixth vein treated if applicable |
| Chemical concentration for foam sclerotherapy of primary vein treated if applicable | Chemical concentration of foam sclerotherapy in % if applicable |
| Chemical concentration for foam sclerotherapy of second vein treated if applicable | Same as above for second vein treated if applicable |
| Chemical concentration for foam sclerotherapy of third vein treated if applicable | Same as above for third vein treated if applicable |
| Chemical concentration for foam sclerotherapy of fourth vein treated if applicable | Same as above for fourth vein treated if applicable |
| Chemical concentration for foam sclerotherapy of fifth vein treated if applicable | Same as above for fifth vein treated if applicable |
| Chemical concentration for foam sclerotherapy of sixth vein treated if applicable | Same as above for sixth vein treated if applicable |
| Volume of foam injected for primary vein treated if applicable | Volume of foam injected into vein in ml if applicable |
| Volume of foam injected for second vein treated if applicable | Same as above for second vein treated if applicable |
| Volume of foam injected for third vein treated if applicable | Same as above for third vein treated if applicable |
| Volume of foam injected for fourth vein treated if applicable | Same as above for fourth vein treated if applicable |
| Volume of foam injected for fifth vein treated if applicable | Same as above for fifth vein treated if applicable |
| Volume of foam injected for sixth vein treated if applicable | Same as above for sixth vein treated if applicable |
| Volume of embolic adhesive injected for primary vein treated if applicable | Volume of embolic adhesive injected into vein in ml if applicable |
| Volume of embolic adhesive injected for second vein treated if applicable | Same as above for second vein treated if applicable |
| Volume of embolic adhesive injected for third vein treated if applicable | Same as above for third vein treated if applicable |
| Volume of embolic adhesive injected for fourth vein treated if applicable | Same as above for fourth vein treated if applicable |
| Volume of embolic adhesive injected for fifth vein treated if applicable | Same as above for fifth vein treated if applicable |
| Volume of embolic adhesive injected for sixth vein treated if applicable | Same as above for sixth vein treated if applicable |
| Number of phlebectomy incisions for primary vein treated if applicable | Number of discrete incisions needed for phlebectomy, not counting the incision for high ligation, if performed; 1) <10, 2) 10-20, 3) >20 |
| Number of phlebectomy incisions for second vein treated if applicable | Same as above for second vein treated if applicable |
| Number of phlebectomy incisions for third vein treated if applicable | Same as above for third vein treated if applicable |
| Number of phlebectomy incisions for fourth vein treated if applicable | Same as above for fourth vein treated if applicable |
| Number of phlebectomy incisions for fifth vein treated if applicable | Same as above for fifth vein treated if applicable |
| Number of phlebectomy incisions for sixth vein treated if applicable | Same as above for sixth vein treated if applicable |

Abbreviation: GSV (great saphenous vein).

**Supplementary Table 3. Post-operative features for machine learning models**

| **Features (n = 15)** | **Definition based on Vascular Quality Initiative Data Dictionary** |
| --- | --- |
| Complications |  |
| None | No post-operative complications |
| Mild allergic reaction requiring local treatment | Mild allergic reaction requiring local treatment (i.e. topical steroids) for symptoms including hives, rash, or itching |
| Severe allergic reaction requiring systemic treatment | Severe allergic reaction requiring systemic treatment for symptoms including difficulty breathing, difficulty swallowing, or anaphylaxis |
| Migraine | Irretractable headache |
| Visual disturbance | Blurred vision, double vision, other unusual change in vision |
| Cough or chest tightness | New symptoms of cough or chest tightness |
| Systemic infection | Infection in the blood stream affecting multiple body systems |
| Pulmonary embolism | Pulmonary embolism detected on imaging |
| Transient ischemic attack | Neurologic deficits persisting < 24 hours |
| Stroke | Neurologic deficits persisting > 24 hours |
| Other complication | Any other significant post-operative complication not listed above |
| Hospital admission required | Planned or unplanned admission to hospital required post-operatively |
| Post-operative compression therapy | Post-operative compression therapy prescribed including stocking, bandage, stocking and/or bandage, or none or not reported |
| Number of days of compression therapy prescribed | Number of days for which treating physician prescribed compression therapy |
| Additional sclerotherapy planned for ipsilateral leg | Additional sclerotherapy planned for ipsilateral leg at a future date |

**Supplementary Table 4. Selection of Extreme Gradient Boosting (XGBoost) model hyperparameters using grid search and cross validation**

| **Hyperparameter** | **Values tested through grid search and cross validation*** | **Optimal value chosen to maximize AUROC** |
| --- | --- | --- |
| Number of rounds | 50, 100, 150, 200, 250, 300, 350, 400, 450, 500 | 250 |
| Maximum tree depth | 2, 3, 4, 5, 6, 7, 8, 9 | 3 |
| Learning rate | 0.4, 0.3, 0.2, 0.1, 0.05, 0.01, 0.001 | 0.05 |
| Gamma | 0, 0.1, 1, 1.5, 2 | 0 |
| Column sample by tree | 0.5, 0.6, 0.7, 0.8, 0.9, 1 | 0.9 |
| Minimum child weight | 1, 3, 5, 7, 10 | 1 |
| Subsample | 0.5, 0.6, 0.7, 0.8, 0.9, 1 | 0.9 |

*Grid search and cross validation are exhaustive methods that iteratively train and evaluate models using every combination of specified hyperparameter values and selects the set of hyperparameter values that optimize model performance.

Abbreviation: AUROC (area under the receiver operating characteristic curve).


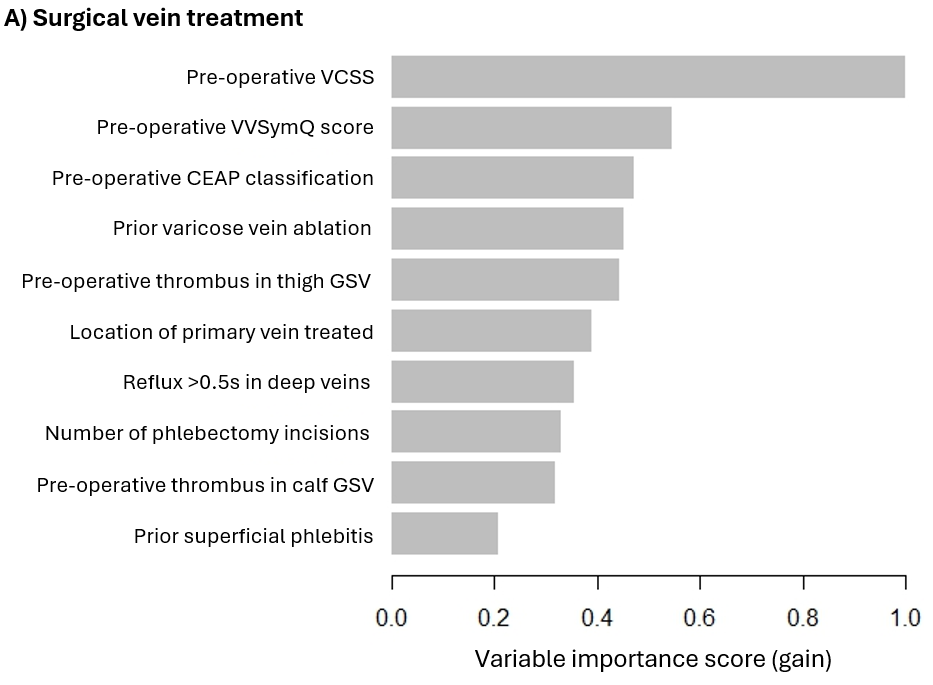


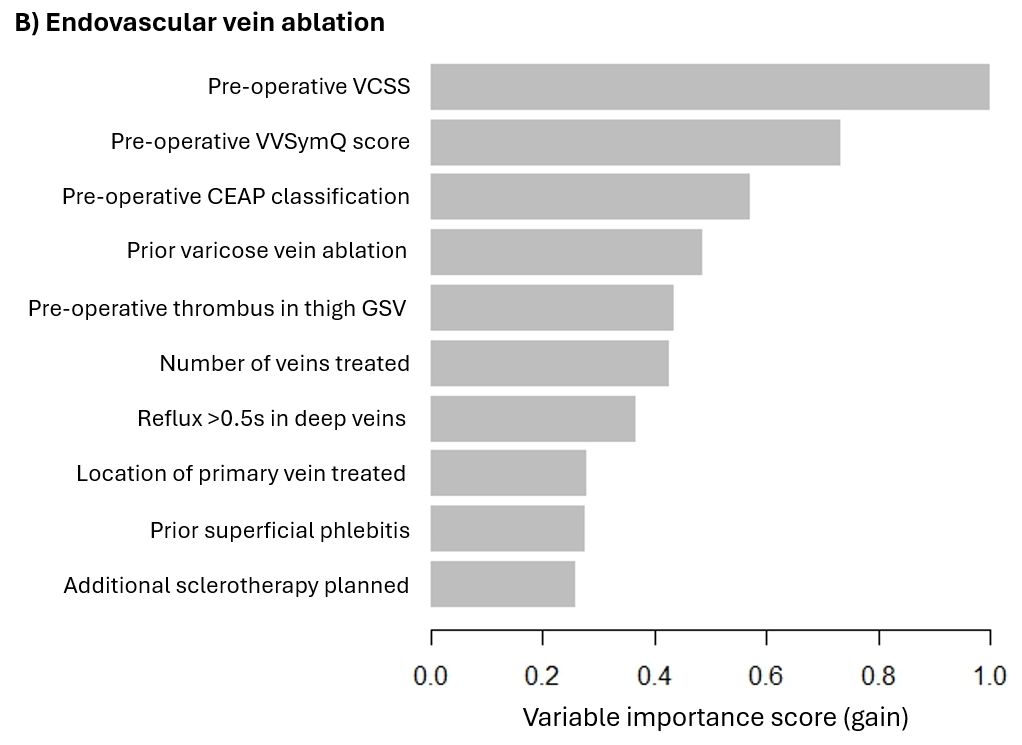


**Supplementary Figure 1. Variable importance scores (gain) for the top 10 predictors of 1-year lack of clinical improvement following varicose vein ablation in the Extreme Gradient Boosting (XGBoost) model at the post-operative stage with subgroup analysis based on A) surgical vein treatment and B) endovascular vein ablation.** Abbreviations: VCSS (venous clinical severity score), VVSymQ (varicose vein symptom questionnaire), CEAP (Clinical-Etiological-Anatomical-Pathophysiological), GSV (great saphenous vein).


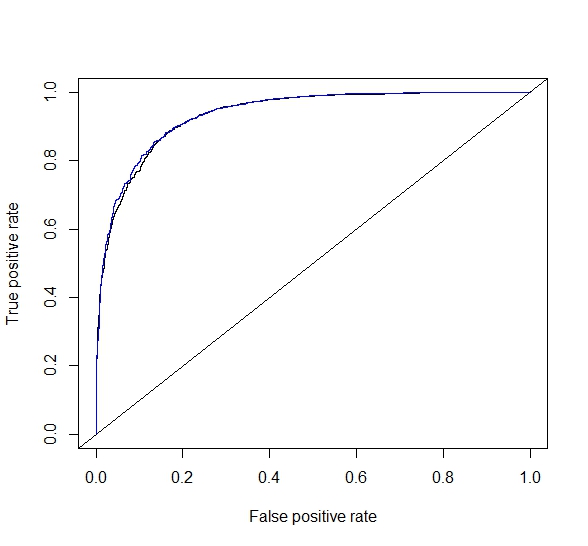


AUROC (95% CI)

Age < 60: 0.94 (0.93 – 0.95)

Age ≥ 60: 0.94 (0.93 – 0.95)

**Supplementary Figure 2. Receiver operating characteristic curve for predicting 1-year lack of clinical improvement following varicose vein ablation using Extreme Gradient Boosting (XGBoost) model at the pre-operative stage with subgroup analysis based on age.** AUROC (area under the receiver operating characteristic curve), CI (confidence interval).


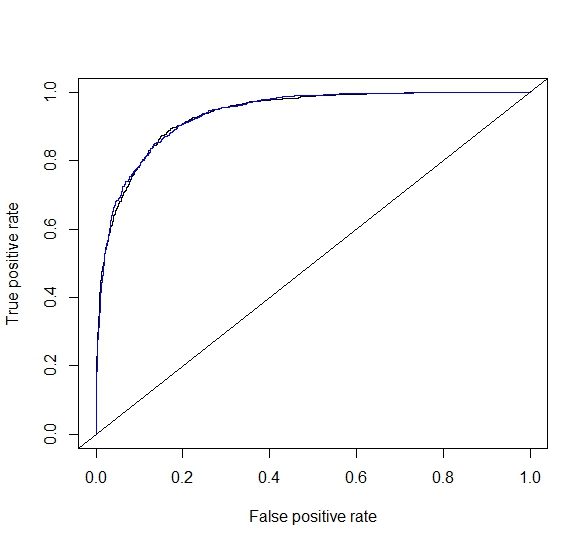


AUROC (95% CI)

Male: 0.94 (0.93 – 0.95)

Female: 0.94 (0.93 – 0.95)

**Supplementary Figure 3. Receiver operating characteristic curve for predicting 1-year lack of clinical improvement following varicose vein ablation using Extreme Gradient Boosting (XGBoost) model at the pre-operative stage with subgroup analysis based on sex.** AUROC (area under the receiver operating characteristic curve), CI (confidence interval).


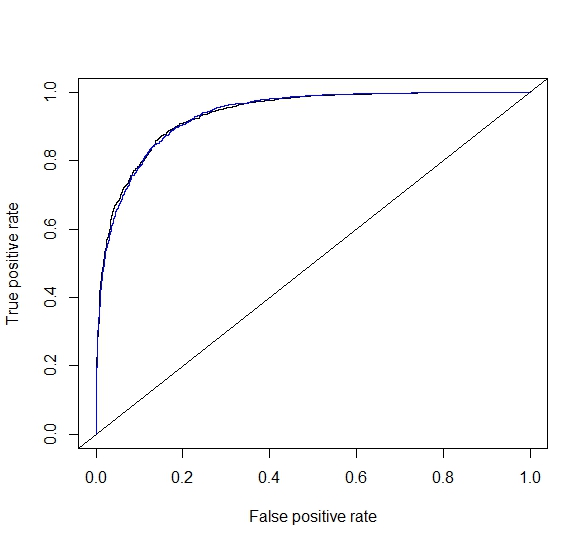


AUROC (95% CI)

White: 0.94 (0.93 – 0.95)

Non-White: 0.94 (0.93 – 0.95)

**Supplementary Figure 4. Receiver operating characteristic curve for predicting 1-year lack of clinical improvement following varicose vein ablation using Extreme Gradient Boosting (XGBoost) model at the pre-operative stage with subgroup analysis based on race.** AUROC (area under the receiver operating characteristic curve), CI (confidence interval).


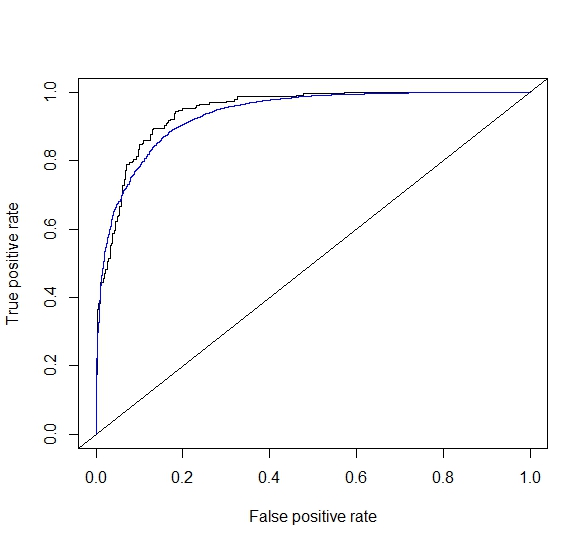


AUROC (95% CI)

Hispanic: 0.95 (0.93 – 0.96)

Non-Hispanic: 0.94 (0.93 – 0.95)

**Supplementary Figure 5. Receiver operating characteristic curve for predicting 1-year lack of clinical improvement following varicose vein ablation using Extreme Gradient Boosting (XGBoost) model at the pre-operative stage with subgroup analysis based on ethnicity.** AUROC (area under the receiver operating characteristic curve), CI (confidence interval).


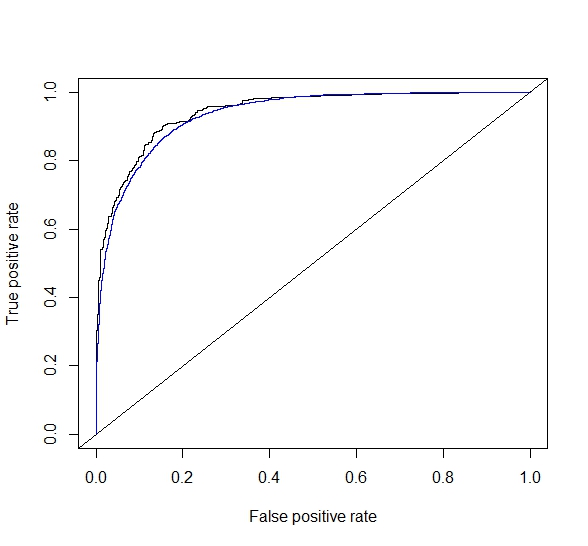


AUROC (95% CI)

Rural: 0.94 (0.93 – 0.96)

Non-rural: 0.93 (0.92 – 0.94)

**Supplementary Figure 6. Receiver operating characteristic curve for predicting 1-year lack of clinical improvement following varicose vein ablation using Extreme Gradient Boosting (XGBoost) model at the pre-operative stage with subgroup analysis based on rurality of residence.** AUROC (area under the receiver operating characteristic curve), CI (confidence interval).


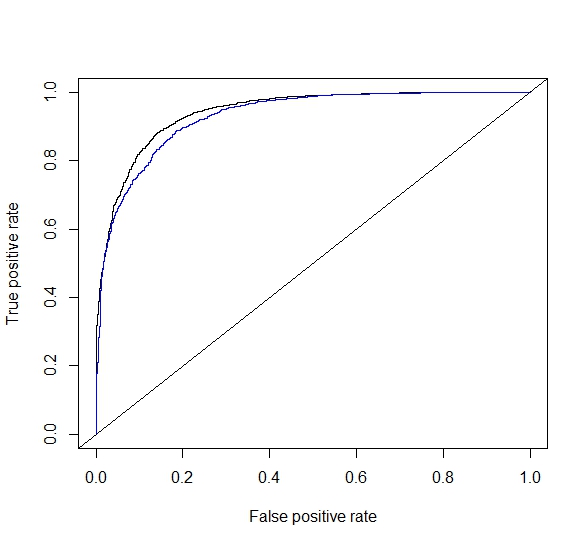


AUROC (95% CI)

ADI ≥ 50%: 0.94 (0.93 – 0.95)

ADI < 50%: 0.94 (0.93 – 0.95)

**Supplementary Figure 7. Receiver operating characteristic curve for predicting 1-year lack of clinical improvement following varicose vein ablation using Extreme Gradient Boosting (XGBoost) model at the pre-operative stage with subgroup analysis based on median Area Deprivation Index (ADI) percentile.** AUROC (area under the receiver operating characteristic curve), CI (confidence interval).


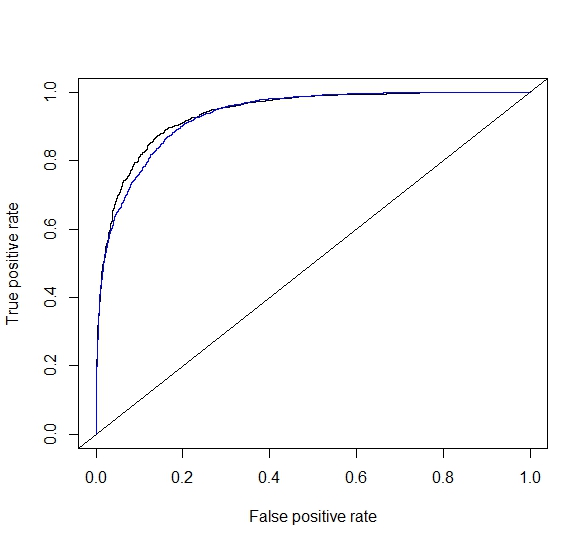


AUROC (95% CI)

Prior ipsilateral varicose vein ablation: 0.94 (0.93 – 0.95)

No prior ipsilateral varicose vein ablation: 0.93 (0.92 – 0.94)

**Supplementary Figure 8. Receiver operating characteristic curve for predicting 1-year lack of clinical improvement following varicose vein ablation using Extreme Gradient Boosting (XGBoost) model at the pre-operative stage with subgroup analysis based on prior ipsilateral varicose vein ablation.** AUROC (area under the receiver operating characteristic curve), CI (confidence interval).


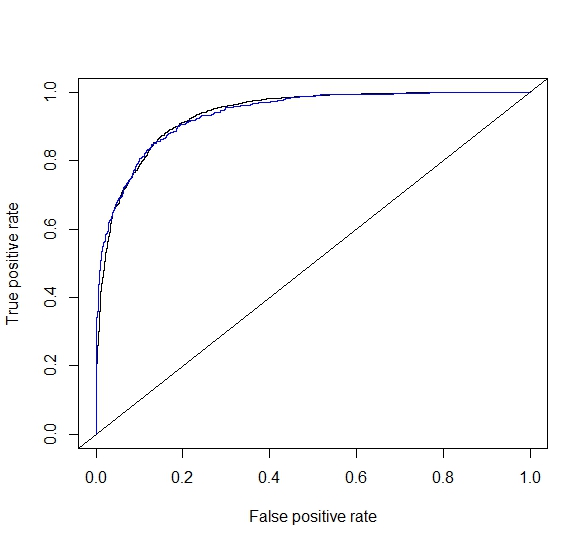


AUROC (95% CI)

Truncal*: 0.94 (0.93 – 0.95)

Non-truncal^: 0.94 (0.93 – 0.95)

**Supplementary Figure 9. Receiver operating characteristic curve for predicting 1-year lack of clinical improvement following varicose vein ablation using Extreme Gradient Boosting (XGBoost) model at the pre-operative stage with subgroup analysis based on location of primary vein treated.** AUROC (area under the receiver operating characteristic curve), CI (confidence interval).

*Truncal veins include the great saphenous vein (GSV), superficial accessory GSV, anterior accessory GSV, and small saphenous vein.

^Non-truncal veins include cluster veins (superficial varicosities) and perforator veins.


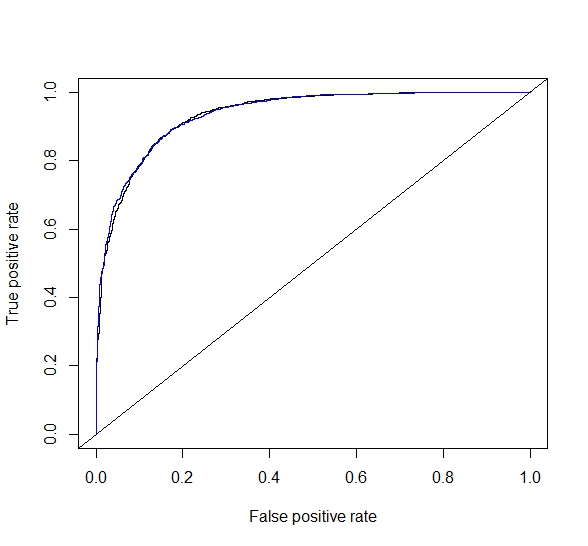


AUROC (95% CI)

Endovenous ablation: 0.94 (0.93 – 0.95)

Surgical treatment: 0.94 (0.93 – 0.95)

**Supplementary Figure 10. Receiver operating characteristic curve for predicting 1-year lack of clinical improvement following varicose vein ablation using Extreme Gradient Boosting (XGBoost) model at the pre-operative stage with subgroup analysis based on treatment type.** AUROC (area under the receiver operating characteristic curve), CI (confidence interval).


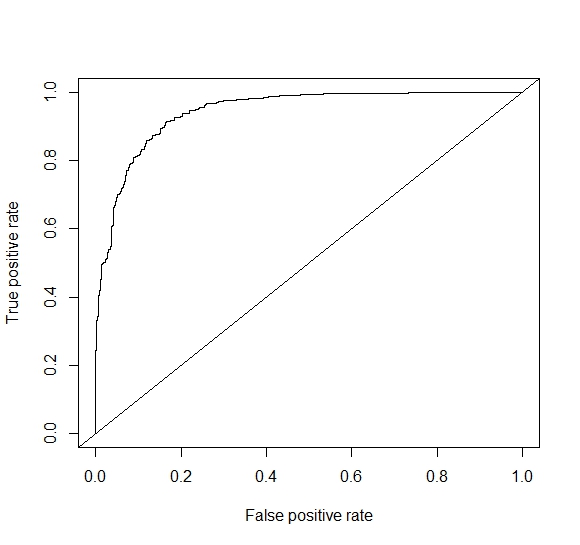


AUROC 0.94 (95% CI 0.93 – 0.96)

**Supplementary Figure 11. Receiver operating characteristic curve for predicting 1-year lack of clinical improvement following varicose vein ablation using Extreme Gradient Boosting (XGBoost) model at the pre-operative stage with subgroup analysis on the last 2 years of data (2023 and 2024).** AUROC (area under the receiver operating characteristic curve), CI (confidence interval).

Secondary performance metrics: accuracy 0.87 (95% CI 0.84 – 0.89), sensitivity 0.90 (95% CI 0.88 – 0.92), specificity 0.84 (95% CI 0.82 – 0.86), positive predictive value 0.84 (95% CI 0.83 – 0.86), negative predictive value 0.90 (95% CI 0.89 – 0.92).
